# Genome-wide characterization of chalcone synthase genes in sweet cherry and functional characterization of *CpCHS1* under drought stress

**DOI:** 10.3389/fpls.2022.989959

**Published:** 2022-08-19

**Authors:** Qiandong Hou, Shuang Li, Chunqiong Shang, Zhuang Wen, Xiaowei Cai, Yi Hong, Guang Qiao

**Affiliations:** ^1^Key Laboratory of Plant Resource Conservation and Germplasm Innovation in Mountainous Region (Ministry of Education), College of Life Sciences/Institute of Agro-bioengineering, Guizhou University, Guiyang, China; ^2^College of Forestry, Institute for Forest Resources & Environment of Guizhou, Guizhou University, Guiyang, China

**Keywords:** sweet cherry, Chinese cherry, chalcone synthase, flavonoid, drought stress

## Abstract

Cherries are one of the important fruit trees. The growth of cherry is greatly affected by abiotic stresses such as drought, which hinders its development. Chalcone synthase (CHS, EC 2.3.1.74) is a crucial rate-limiting enzyme in the flavonoid biosynthetic pathway that plays an important role in regulating plant growth, development, and abiotic stress tolerance. In the current study, three genes encoding chalcone synthase were identified in the genome of sweet cherry (*Prunus avium* L.). The three genes contained fewer introns and showed high homology with CHS genes of other Rosaceae members. All members are predicted to localize in the cytoplasm. The conserved catalytic sites may be located at the Cys163, Phe214, His302, and Asn335 residues. These genes were differentially expressed during flower bud dormancy and fruit development. The total flavonoid content of Chinese cherry (*Cerasus pseudocerasus* Lindl.) was highest in the leaves and slightly higher in the pulp than in the peel. No significant difference in total flavonoid content was detected between aborted kernels and normally developing kernels. Overexpression of Chinese cherry *CpCHS1* in tobacco improved the germination frequency of tobacco seeds under drought stress, and the fresh weight of transgenic seedlings under drought stress was higher than that of the wild type, and the contents of SOD, POD, CAT, and Pro in OE lines were significantly increased and higher than WT under drought stress. These results indicate cherry CHS genes are conserved and functionally diverse and will assist in elucidating the functions of flavonoid synthesis pathways in cherry and other Rosaceae species under drought stress.

## Introduction

Sweet cherry (*Prunus avium* L.) is a member of the Rosaceae family and is one of the most popular fruits. Similarly, the Chinese cherry (*Cerasus pseudocerasus* Lindl.) is an economically important fruit tree and is widely distributed in the southwestern region of China (Zhang et al., 2018). After a long period of selection, a variety named “Manao Hong” Chinese cherry was selected. Which has suitable for cultivation in the mountainous terrain of Guizhou province (Zhang et al., 2012). Guizhou is located on the Yunnan-Kweichow Plateau, and the entire province is composed of mountains and hills. Few economically important fruit trees are suitable for planting here because of the lack of sunshine and rainfall all year round. After years of selective breeding, “Manao Hong” cherry has been able to grow in harsh environments. However, under the environment of uneven annual rainfall and karst landforms in Guizhou, cherries still face the impact of drought.

Flavonoids are a kind of phenolic compound, which are secondary metabolites widely synthesized in terrestrial plants, and are important nutrients in fruits and food crops (Hernández et al., 2009; Shen et al., 2022). Flavonoids perform crucial functions in plant growth, development, environmental adaptation, and response to biotic or abiotic stresses (Petrussa et al., 2013). Chalcone synthase (CHS) is a crucial rate-limiting enzyme in the flavonoid biosynthetic pathway that catalyzes the condensation of malonyl-CoA and ρ-coumaroyl-CoA to produce naringenin chalcone, which serves as the precursor of a variety of flavonoid derivatives (Winkel-shirley, 2001; Petrussa et al., 2013). The molecular weight of the CHS protein is 42–45 kDa, and modeling of the three-dimensional structure shows that there are four highly conserved amino acid residues (Cys, Phe, His, and Asn) in the center of the CHS molecule, which is the core of the catalytic function of CHS (Pandith et al., 2020). Based on their function and structure, CHS or CHS-like proteins are type III polyketide synthase (PKS) and have been extensively studied in diverse species (Flores-Sanchez and Verpoorte, 2009; Pandith et al., 2020). Chalcone is an intermediate product of the flavonoid synthesis pathway, not the final product. For example, flavanones and dihydro flavanols synthesized from chalcone as the substrates will eventually form anthocyanins and catechins (Singh et al., 2017). These flavonoids are involved in the response to and protection of plants from abiotic and biotic stress, including ultraviolet radiation, temperature, humidity, and pathogen attack (Fini et al., 2011).

Chalcone synthases in plants are a multi-member gene family. Owing to the availability of plant genomes, this family has been systematically identified in numerous species, e.g., eight members in wheat (Glagoleva et al., 2019), five in phalaenopsis (Kuo et al., 2019), 20 in cotton (Kong et al., 2020), 14 in maize (Han et al., 2016). The evolution and function of these genes have been studied to a certain extent, which has greatly broadened the understanding of the function of this gene family in plants. In citrus, transcription of CHSs was enhanced by methyl jasmonate (MeJA) and led to the accumulation of flavonoids (Wang et al., 2018). As for gerbera, only *GCHS4* correlated with flavonoid biosynthesis, while GcCHS4worked for anthocyanin (Deng et al., 2014). Recently, an involvement of CHS in anthraquinone biosynthesis was reported in *Senna tora*, and further analysis showed a species-specific expansion of CHSs in this grass (Kang et al., 2020). The effects of drought on CHS gene expression have been explored somewhat in horticultural plants. After UV-B and drought treatments, the expression of the CHS gene significantly increased in the chili pepper (*Capsicum annuum* L.), indicating that this gene’s function had changed (Rodríguez-Calzada et al., 2019). In addition, proteomic data showed that CHSs were found downregulated in tea plants under drought stress along with accumulated flavonoids (Gu et al., 2020). The aforementioned evidence suggests that CHS is essential for drought stress adaption.

Drought is an important environmental factor, that greatly affects plant growth and development, and is a major limitation to agricultural production. The accumulation of phenolics can increase the drought tolerance of plants (Sharma et al., 2019). The direct result of drought stress is the reduction in water uptake from the soil; the effect within cells is the escape of electrons from the mitochondria, which increases the reactive oxygen species (ROS) concentration in the cell, and thereby weakens the antioxidant capacity of the cell (Choudhury et al., 2013; Sachdev et al., 2021). To adapt to this abiotic stress, plants have the ability to scavenge oxygen free radicals. Previous researches have shown that flavonoids can scavenge reactive oxygen species produced by plants under abiotic stress, thereby enhancing plant resistance (Ma et al., 2014; Naing and Kim, 2021). Drought can induce the up-regulation of CHS gene expression, thereby increasing the drought tolerance of plants (Chen et al., 2017; Wang et al., 2017a).

One goal in breeding agricultural crops is to increase their ability to tolerate drought, which can also enhance production safety precautions. CHS genes play a positive role in plant drought resistance but have been rarely studied in cherries. In the current study, we analyzed the genome of sweet cherry to identify genes encoding chalcone synthase and explored the molecular characteristics of these genes. In addition, we isolated a gene encoding chalcone synthase from the Chinese cherry “Manao Hong” and analyzed its function in transgenic tobacco under drought treatment. The purpose of this study was to investigate the response to drought stress of tobacco overexpressing the Chinese cherry CHS gene, and to provide a reference for future studies of the molecular mechanism of drought tolerance in Chinese cherry.

## Materials and methods

### Plant material

The experimental material is a 2-year-old cherry plant grown in a rain shelter (out annual average temperature is 15°C; greenhouse day/night: 20–23°C/15–18°C; the average annual relative humidity is 77%; colorless transparent plastic film). The root system was cleaned, immediately frozen in liquid nitrogen, and stored at −80°C for later use. During the ripening period of “Manao Hong” cherry, the mature fruits and leaves were collected, and the peel and pulp were separated from the fruits, while the normally developed kernels and the aborted kernels are collected and set aside. The tobacco used in this experiment was *Nicotiana benthamiana*. Tobacco seeds were sterilized with 75% ethanol and 10% NaClO, sown on Murashige–Skoog (MS) medium, and vernalized for two days at 4°C. Subsequently, the seedlings were grown for 14 days under the conditions of 50% relative humidity and a 14 h/10 h (day/night) photoperiod to generate sterile tobacco seedlings.

### Sweet cherry CHS identification and analysis

The sweet cherry genome (Tieton Genome v2.0) was downloaded from the GDR database^1^ (Wang et al., 2020b). The sweet cherry genome was searched using *Arabidopsis thaliana* CHS as the query sequence with the BLAST method. The protein molecular weight (MW) and theoretical isoelectric point (pI) were calculated with the ExPASy tool.^2^ Prediction of subcellular localization is based on previous research (Sohail et al., 2022). Motifs were predicted using the MEME Suite.^3^ The gene structure was visualized using genome annotation information and TBtools (Chen et al., 2020). The candidate sequences were submitted to Pfam^4^ for verification. CHS protein sequences of common species of Rosaceae, Brassicaceae, Solanaceae, and Poaceae were downloaded from the NCBI and UniProt^5^ database. A multiple sequence alignment was generated using Clustal W (Li, 2003). The protein three-dimensional structure was predicted using SWISS-MODEL^6^ and PHYRE2.^7^ Use Pymol software to predict the active site of the protein model, and the active site refers to previous research reports (Imaizumi et al., 2020). The phylogenetic tree was constructed by setting 1,000 bootstrap, Poisson model and other default parameters with MEGA11 (Tamura et al., 2021), and visualized with the online tool ChiPlot.^8^ The RNA-seq data of PRJNA255452 (Wei et al., 2015), PRJNA369332 (Ionescu et al., 2017), and PRJNA550274 (Bing et al., 2020) were downloaded from NCBI-SRA and mapped to the sweet cherry genome after quality control. The gene expression level was arranged using the pipeline of HISAT, StringTie, and Ballgown (Fan et al., 2021). The expression level was calculated with the log2^(FPKM + 1)^ function using TBtools.

### RNA isolation and gene cloning

Total RNA was extracted from the Chinese cherry root using an RNA kit (OMEGA, China). The quantity and purity of the isolated RNA were checked using a Nano Drop 2000 spectrophotometer (Thermo Fisher Scientific, Waltham, MA, United States). The first strand of cDNA was synthesized using the PrimeScript™ RT reagent Kit with gDNA Eraser (TaKaRa, Dalian, China). For the coding sequence of *CpCHS1*, full-length amplification primers were designed using Primer5, and qRT-PCR primers were generated using the RNA-seq sequences as a reference. The primers used are listed in Supplementary Table S1. The full-length coding frame of the Chinese cherry CHS gene was amplified by PCR, and sequenced after transformation into *E. coli.* The correctly sequenced gene was designated *CpCHS1*.

### Vector construction and genetic transformation

The coding sequence of *CpCHS1* was inserted into the plant expression vector pBWA(V)KS to produce the *CpCHS1*-35S construct driven by the *Cauliflower mosaic virus* (CaMV) 35S promoter. The DNA recombinant plasmid was transfected into *Agrobacterium tumefaciens* strain GV1301. Genetic transformation was conducted using the method of inducing plantlet regeneration of tobacco from leaf explants after transformation (Sunilkumar et al., 1999). Tobacco leaves pre-cultured for 2 weeks were cut into 0.5 cm size, surface sterilized, and washed 5 times with sterile water. After pre-culturing in MS medium for 2 days in the dark, using *Agrobacterium tumefaciens* (OD600 = 0.5) for 10 min of infection. Then, it was plated on MS medium, cultured at 25 ± 2°C for 3 days, and then transferred to MS medium containing kanamycin (Kana). After screening *CpCHS1*-positive tobacco plants with Kana, genomic DNA was extracted using a DNA extraction kit (TIANGEN, Beijing, China) for PCR verification with primers listed in Supplementary Table S1.

### Assays for drought treatment

Analysis of germination rate of tobacco seeds under drought. Transgenic lines and wild-type (WT) seeds were sterilized and sown in 1/2 MS medium supplemented with 100, 200, and 300 mm mannitol. Among them, the seeding of different strains on 1/2 MS medium lacking mannitol was used as the control. After sowing, the petri dishes were incubated in a tissue culture room at 23 ± 2°C under a 16 h/8 h (day/night) photoperiod. The germination frequency of transgenic and WT seeds in each treatment was counted after 14 days.

Drought tolerance analysis of tobacco plantlets. The OE lines and wild-type tobacco seeds were sterilized and sown on 1/2 MS supplemented with 50 mg/l Kana or lacking Kana, and incubated at 23 ± 2°C with a 16 h /8 h (day/night) photoperiod in the tissue culture room for 7 days. Transgenic and wild-type tobacco seedlings with the same growth vigor were selected and transferred to 1/2 MS medium supplemented with 100, 200, and 300 mm mannitol, and the growth status of different lines on 1/2 MS medium was used as a control for 14 days treatment. The growth status of transgenic and wild-type tobacco seedlings in each treatment with different concentrations of mannitol was observed, and the root length and fresh weight were measured.

Phenotypic analysis of tobacco under drought treatment. Seeds of *CpCHS1* overexpression (OE) lines and wild-type tobacco were sterilized and sown on 1/2 MS supplemented with 50 mg/l Kana or 1/2 MS solid medium. The seeds were incubated at 4°C for 2 days, and then incubated at 23 ± 2°C for 16 /8 h (day/night) for 14 days. The seedlings were transplanted into soil, and after 4 weeks, seedlings of similar growth were selected for natural drought stress treatment. Watering was withheld for 15 days. The morphological changes of the transgenic tobacco and wild-type tobacco were observed and recorded at 0, 5, 10, and 15 days of natural drought treatment and after rehydration for 7 days, and the survival percentage and growth rate of the plants were determined.

All experiments were replicated thrice and T3 generation of tobacco plants was used as experimental material.

### Measurement of indices of drought tolerance

Peroxidase (POD), Superoxide Dismutase (SOD), Malondialdehyde (MDA), Catalase (CAT), and Proline (Pro) Content in the transgenic and WT plants were measured as previously described (Hou et al., 2022). Fresh samples were taken and disrupted using sonication (Power 200 W, ultrasonic for 3 s, interval of 10 s, repeat 30 times). After extraction POD, SOD, MDA, and Pro according to the instructions, were measured by visible light spectrophotometry, and the absorbance was measured at 470 nm, 560 nm, and 520 nm, respectively. Likewise, cells from fresh samples were disrupted by sonication, and CAT content was determined using UV spectrophotometry (240 nm). Three biological replicates were set up for each experiment. The Micro Plant Flavonoids Assay Kit (Spectrophotometer/Microplate Reader; Solarbio, Beijing, China) was used to determine the total flavonoid content in transgenic plants in accordance with the manufacturer’s instructions.

### Expression pattern of related genes in transgenic plants

The expression levels of the core genes *PAL*, *C4G*, *4CH*, and *CHI* in the flavonol synthesis pathway in response to drought treatment were analyzed. The primers reported in a previous study were used (Lijuan et al., 2015). Total RNA was isolated from drought treatment WT and OE lines tobacco at different time points with the RNA Kit (OMEGA, China). Quantitative real-time PCR reactions were performed on the CFX ConnectTM Real-Time System (BIO-RAD, Hercules, CA, United States) using SYBR Mix (Applied Biosystems, Shanghai, China).

### Statistical analysis

All experiments were conducted with three biological replicates, and three technical replicates were analyzed for each sample. The significance of differences between means was determined with Duncan’s multiple range test using SPSS 20.0 at the 5% significance level.

## Results and discussion

### Sweet cherry CHS gene characteristic

**Figure 1:**
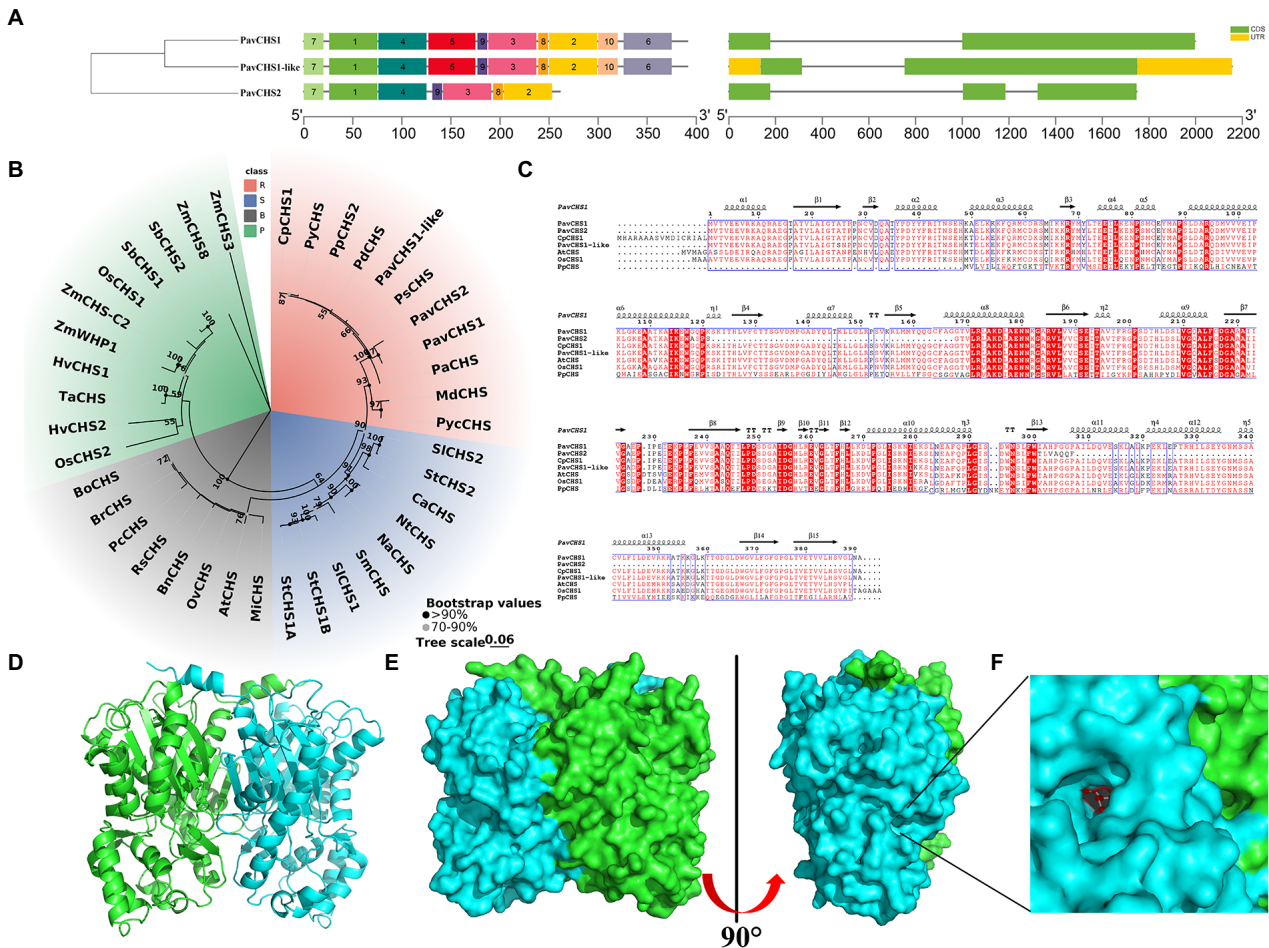
Characterization of CHS in sweet cherries. **(A)** Conserved motifs (left) and gene structures (right) of PavCHSs, conserved motifs and gene structures are represented by square boxes with different colors, and the black lines on the right represent introns. **(B)** The evolutionary relationship of CHS genes in four types of plants, different types of plants are represented by different colored branches on the phylogenetic tree. R, S, B, and P represent Rosaceae, Solanaceae, Brassicaceae, and Poaceae, respectively. **(C)** Multiple sequence alignment of CHS genes in sweet cherry, Chinese cherry, *Arabidopsis thaliana*, rice, and peach; red background indicates complete agreement, above the sequence is the predicted protein secondary structure. **(D)** Protein tertiary structure of PavCHS1. **(E)** The surface structure of PavCHS1 protein. **(F)** The possible active site of PavCHS1. Cp (*Cerasus pseudocerasus*); Py (*Prunus yedoensis*); Pp (*Prunus persica*); Pd (*Prunus dulcis*); Pav (*Prunus avium*); Ps (*Prunus salicina*); Pa (*Prunus armeniaca*); Md (*Malus x domestica*); Pyc (*Pyrus communis*); Sl (*Solanum lycopersicum*); St (*Solanum tuberosum*); Ca (*Capsicum annuum*); Nt (*Nicotiana tabacum*); Na (*Nicotiana alata*); Sm (*Solanum melongena*); Mi (*Matthiola incana*); Bn (*Brassica napus*); Rs (*Raphanus sativus*); Pc (*Pugionium cornutum*); Br (*Brassica rapa*); Bo (*Brassica oleracea*); Os (*Oryza sativa*); Hv (*Hordeum vulgare*); Ta (*Triticum aestivum*); Zm (*Zea mays*); Sb (*Sorghum bicolor*).

The BLAST search of the sweet cherry genome detected, three genes encoding chalcone synthase, which were designated *PavCHS1*, *PavCHS1-like*, and *PavCHS2* (Supplementary Table S2). Compared with transcription factor gene families such as MYB, the CHS gene family contains fewer members. For example, three CHS members are known in apple (Yahyaa et al., 2017), 5 members in mulberry (Wang et al., 2017a), 7 members in eggplant (Wu et al., 2020), 8 genes in wheat (Glagoleva et al., 2019), and 9 genes in soybean (Yi et al., 2010). The proteins encoded by the three *PavCHS* genes were 261 or 391, the molecular weights ranged from 28755.09 to 42778.47, and the theoretical isoelectric points were all less than 7. Thus, each gene encodes a hydrophilic protein (Supplementary Table S3). The three genes were located on chromosome 1 and are relatively close to each other. The subcellular localizations of all members were predicted to be in the cytoplasm (Supplementary Table S3), suggesting that these genes function in the cytoplasm. Analysis of the gene structure revealed that the *PavCHS* genes contained one or two introns (Figure 1A), with fewer introns, indicating that the CHS genes may have fewer sequence insertions in sweet cherry and appeared later in evolution (Liu et al., 2021b). The paucity of introns also indicates that the function of the genes may be limited or relatively specific, and the genes are more likely to be induced by external stress (Hanada et al., 2008). The presence of introns may also be associated with gene expression, as some introns have “intron-mediated enhancement’ sequences” that can significantly affect gene expression (Anireddy and Maxim, 2008; Shaul, 2017). A search with the MEME tool revealed 10 conserved motifs, among which *PavCHS2* lacked Motif 5, Motif 6, and Motif 10 (Figure 1A).

To explore the homology and evolution of the PavCHS and CpCHS1 proteins, relevant protein sequences for other members of the Rosaceae, Solanaceae, Brassicaceae, and Poaceae were downloaded and a multiple sequence alignment was generated (Figure 1B). The PavCHS proteins and *CpCHS1* were phylogenetically similar to proteins from other *Prunus* species, suggesting that these genes may have high homology. Interestingly, *PavCHS1* and *PavCHS1-like* are highly similar, with only 3 amino acid differences. The two genes may be derived from the same ancestral gene or have similar functions. Similar gene members include *SlMADS53* and *SlMADS54* in the MADS-box family of tomato (Wang et al., 2019), and *PmbHLH20* and *PmbHLH21* in *Prunus mume* (Wu et al., 2022). The four families included in this analysis were evolutionarily homogenized into one class, which indicated that the evolutionary relationships of CHS genes may be basically consistent with diversification in the families. CHS genes are present in bryophytes, which may represent the earliest CHS genes and have differentiated functional differences (Jiang et al., 2006). Studies have shown that the pro-core of the catalytic cysteine of the CHS gene has been gradually enhanced during the diversification of vascular plants (Liou et al., 2018). These changes in CHS have increased the diversity of flavonoid biosynthesis, which has important implications for plant survival in terrestrial ecology (Durbin et al., 2000). The multiple sequence alignment showed that the CHS sequences of rice, *Arabidopsis*, and cherry were highly similar, suggesting that the CHS gene is highly conserved in vascular plants (Figure 1C).

Previous studies have shown that CHS proteins form homodimers. In the current study, a protein homology model was constructed in SWISS-MODEL and PYRE2 using the protein sequence of PavCHS1. The results showed that the PavCHS1 protein has a homodimer structure (Figure 1D), which is highly similar to *GmCHS1* in soybean (Imaizumi et al., 2020). In the dimeric structure of *MsCHS*, the active site is located at the intersection of a specialized “CoA-binding tunnel” and a large internal “initiation/extension/cyclization cavity”; this site is deeply buried in both monomers; and each monomeric active site contains a catalytic triad of Cys164, His303 and Asn336 residues at the top of the active site cavity (Abe and Morita, 2010). In this present study, *PavCHS1* contained such an active site, which was predicted to be at Cys163, Phe214, His302, and Asn335 of PavCHS1 (Figures 1E,F). In the three-dimensional structure, the catalytic triad and Phe216 intersect with three interconnected cavities, comprising the CoA-binding tunnel, the coumaroyl-binding pocket, and the cyclization pocket, forming the active site structure of CHS (Pandith et al., 2020). At the beginning of the synthesis reaction, Cys164 nucleophilically attacks the thioester carbonyl group, resulting in the transfer of the coumaroyl moiety to the cysteine side chain. This process is maintained by the sulfate anion of Cys164 through ionic interaction with the imidazolium cation of His303. The His303 and Asn336 residues form hydrogen bonds with the thioester carbonyl group, which further stabilized the formation of tetrahedral reaction intermediates. Coenzyme dissociates from the enzyme, leaving a coumaroyl thioester at Cys164 (Jez et al., 2001; Abe and Morita, 2010; Imaizumi et al., 2020).

### Expression profile of *PavCHSs* and flavonoids content in Chinese cherry fruit

**Figure 2:**
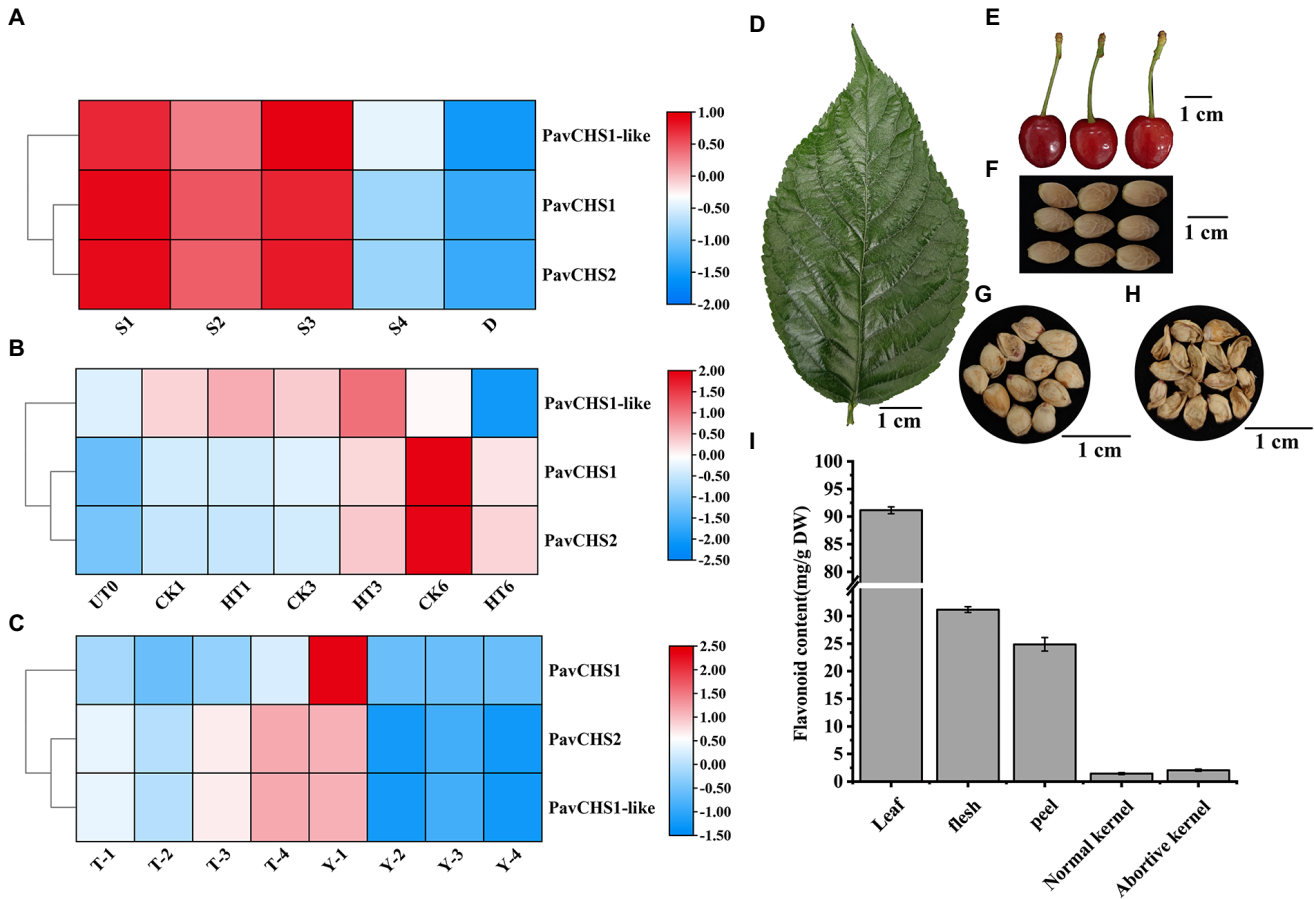
RNA-seq based expression patterns of *PavCHS* and flavonoid content in different tissues of Chinese cherries. **(A–C)** Expression patterns of *PavCHS* genes in RNA-seq of public databases; where **(A–C)** are flower bud differentiation, flower bud processing and fruit development, respectively. **(D–H)** Leaf and fruit tissues of Chinese cherries at maturity, scale bar is 1 cm. **(I)** Determination of flavonoid content in Chinese cherries.

To examine the expression pattern of the *PavCHS* genes, their expression at different stages of fruit development and floral bud differentiation was explored from public data. The expressions of the three *PavCHS* genes were gradually downregulated during floral bud differentiation, indicating that these genes may play an important role in the early stage of floral bud differentiation (Figure 2A). *PavCHS1* and *PavCHS2* were significantly inhibited on day 6 of cyanamide-treated flower buds, suggesting that their functions were altered (Figure 2B). During fruit development of the sweet cherry cultivar “Tieton,” *PavCHS1-like* and *PavCHS2* were gradually up-regulated with fruit ripening; in the cultivar “13–11,” the three CHS genes were highly expressed in the initial period of fruit development, and thereafter were downregulated expression (Figure 2C). These results indicated that CHS genes in sweet cherry may have multiple functions. In Paeonia, *PhCHS* is up-regulated in the first two stages of petal development and thereafter is downregulated which may be the result of ubiquitination (Gu et al., 2019). The expression of chalcone synthase and related genes was proportional to the population dynamics of pine wood nematode, and these genes dominated the co-expression module, suggesting that CHS and related genes play an important role in the response of pine to suppression of pine wood nematode infection (Chen et al., 2021). The three CHS genes in apple are highly expressed in young leaves and also in the fruit skin (Yahyaa et al., 2017).

Flavonoids are beneficial nutrients and play crucial roles in the maintenance of the biological activities of plants. In the current study, the total flavonoid content of flavonoids in the pericarp and pulp of Chinese cherry fruit at the ripe stage, and in the leaves at the ripe stage of the fruit, was determined (Figures 2D,E). In addition, during cherry fruit development, various factors may lead to embryo abortion, which can lead to fruit abscission or poor fruit development (Deng et al., 2019; Qiu et al., 2020). To explore whether embryo abortion was associated with the flavonoid content, the flavonoid content in normal and aborted kernels of Chinese cherry fruit was also determined (Figures 2F–H). The flavonoid content was highest in the leaves, and that in the pulp was slightly higher than that in the peel (Figure 2I). There was no statistically significant difference in flavonoid content between normally developed and aborted kernels, suggesting that abortion may not be associated to flavonoid levels. According to studies, pollen abortion may be related to the flavanone synthesis pathway, and male sterility and cytoplasmic male sterility are both related to and necessary for the suppression of CHS or other flavonoids’ biosynthetic gene expression (Yang et al., 2008). *GbCHS06, GbCHS10, GbCHS16*, and *GbCHS19* in cotton were abnormally expressed in abortion pollen (Kong et al., 2020). Although no difference in total flavonoid content was detected in normal and aborted kernels of Chinese cherry, further study is required to resolve the mechanism of kernel abortion. The expression of CHS gene in satsuma mandarin (*Citrus unshiu* Marcow) was gradually downregulated during fruit ripening, accompanied by a decrease in total flavonoid content, indicating that the expression of CHS gene was positively correlated with flavonoid content (Wang et al., 2010). The increase of CHS gene in *Silybum marianum* was positively correlated with the content of silybin; and *CHS1*, *CHS2*, and *CHS3* were involved in the biosynthesis of silybin, and the transcription levels of these three genes were increased under light and salt treatments (El-Garhy et al., 2016). These results demonstrate the diversity of CHS gene functions.

### Overexpression of *CpCHS1* enhances seed germination in drought stress

**Figure 3:**
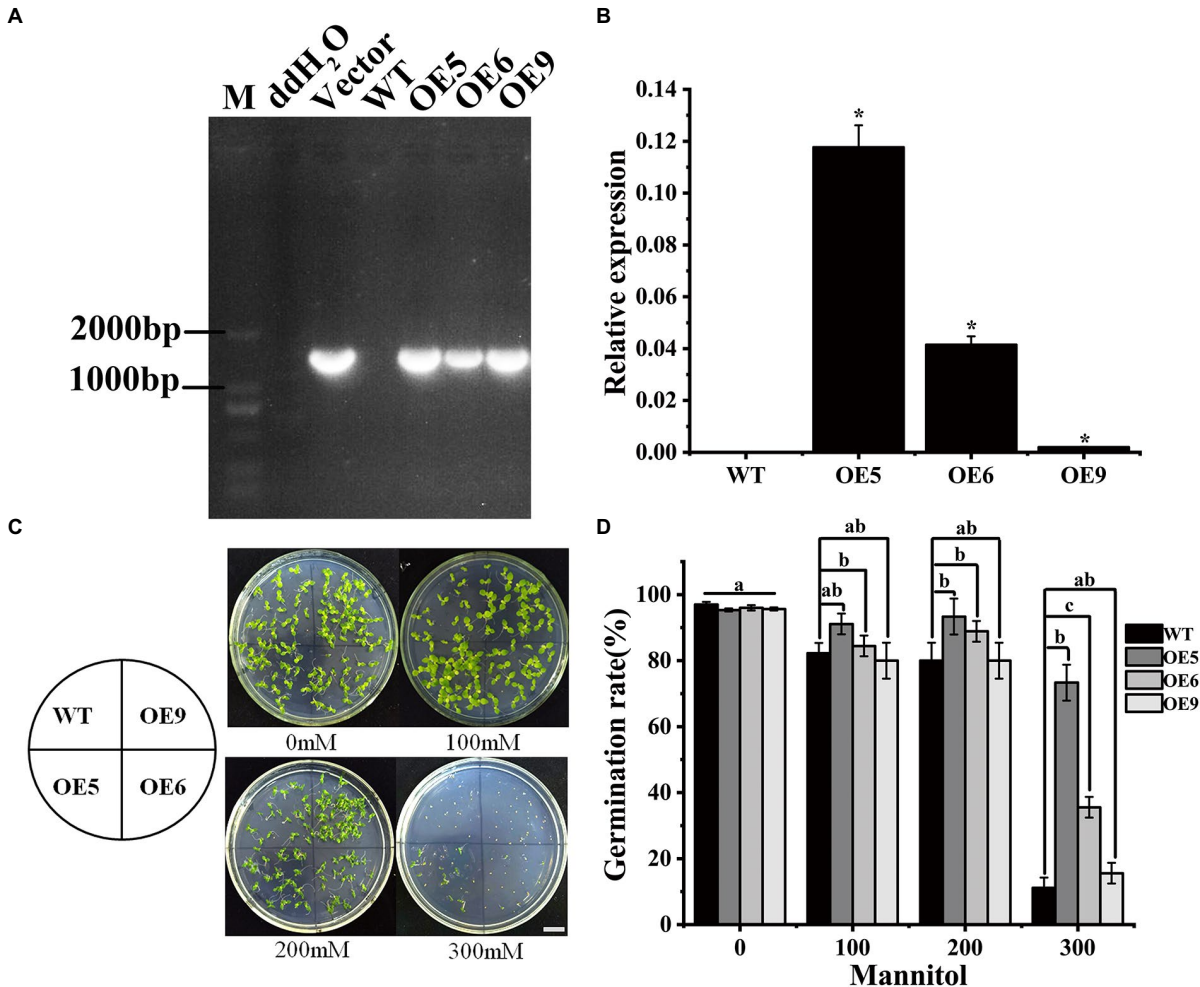
Genetic transformation of Chinese cherry *CpCHS1* into tobacco. **(A)** PCR method to identify transgenic tobacco lines. **(B)** Detection of the expression level of *CpCHS1* in tobacco. **(C)** Germination of WT and OE lines at different mannitol concentrations. **(D)** Statistics on the germination rate of tobacco seeds under different mannitol concentrations. Different letters and ‘*’ indicate the significant differences of the three replicates as determined by SPSS software (*p* < 0.05).

To evaluate the biological function of CHS genes in Chinese cherry, *CpCHS1* was cloned (Supplementary Table S2). The coding sequence of *CpCHS1* was 1,227 bp. Heterologous overexpression of CpCHS1 in tobacco resulted in the identification of three tobacco OE lines (Figure 3A). Among the three OE lines, the expression level was highest in OE5 (Figure 3B). The response of plants to drought stress is an extremely complex process involving changes in physiology, phenotype, and gene regulation (Zia et al., 2021). CHS genes are involved in the complex regulatory network in response to drought stress (Kubra et al., 2021). In the current study, the seed germination frequency differed among the tobacco OE lines in a mannitol-containing medium, with OE5 having the highest seed germination rate (Figures 3C,D). Compared with the WT, the seed germination rate of the OE line was higher than that of the wild type. These results indicated that overexpression of *CpCHS1* could enhance the germination of tobacco seeds under drought conditions. With regard to the salt tolerance of *Populus euphratica*, genes associated with the flavonoid synthesis pathway are differentially expressed, illustrating the importance of flavonoid-related genes in participating in stress responses to abiotic stress (Zhang et al., 2019). The improvement of plant tolerance to drought and salt stress is related to the increase of flavonoids, phenolics, and alkaloids (Resmi et al., 2015). Similarly, overexpression of *CpCHS1* increased the germination frequency of tobacco under experimentally induced drought stress, possibly by increasing flavonoid or alkaloid biosynthesis (Gu et al., 2020; Yang et al., 2020).

**Figure 4:**
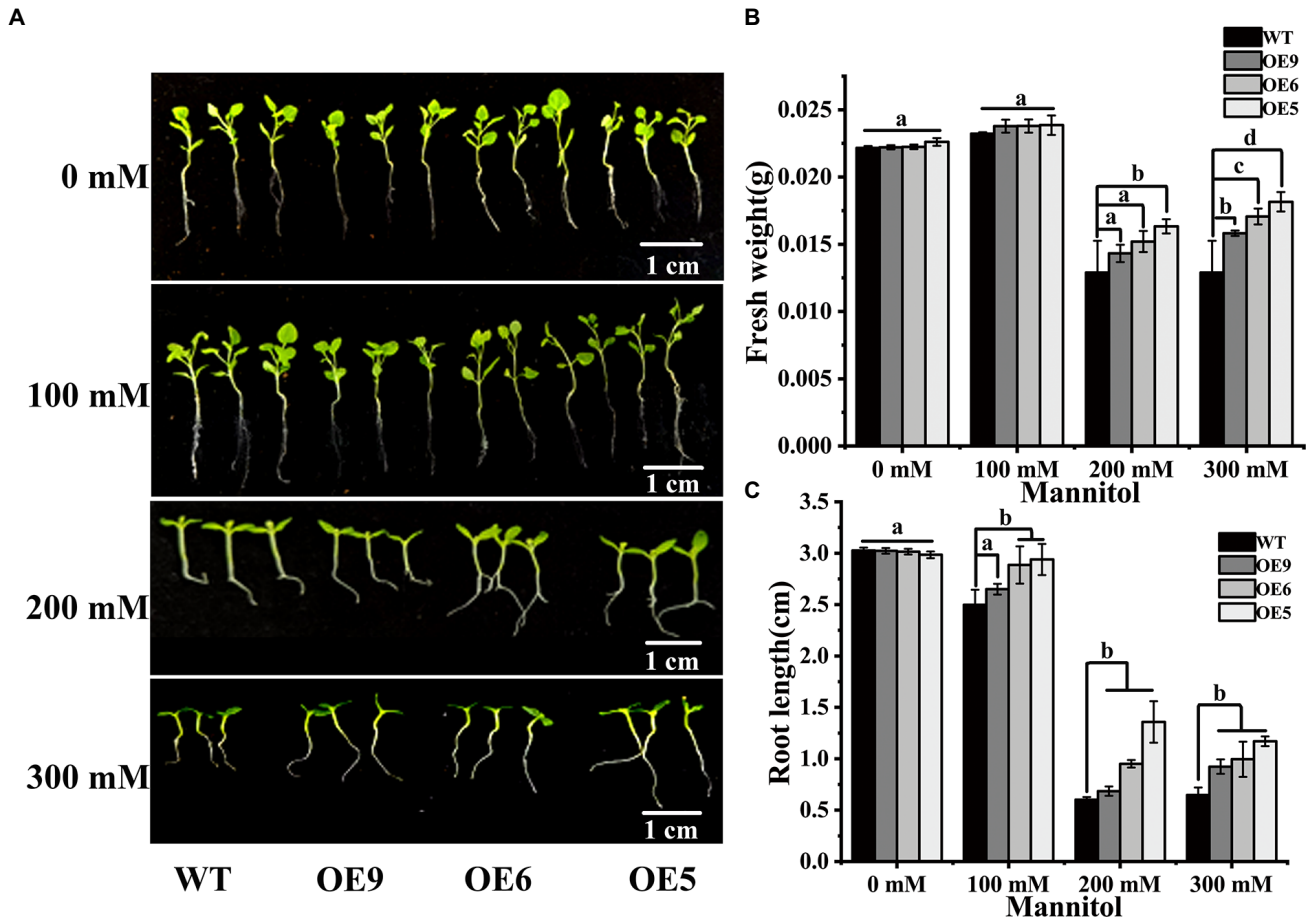
WT and OE line tobacco seedlings were treated with different mannitols. **(A)** Phenotypic comparison after 14d treatment with different mannitol. **(B)** Fresh weight statistics after treatment in WT and OE lines. **(C)** Root length statistics after treatment in WT and OE lines. Different letters indicate the significant differences of three replicates as determined by SPSS software (*p* < 0.05).

Overexpression in tobacco of a quinolone synthase gene, a participant in the flavonoid synthesis pathway, of bael increased the root length of the transgenic plants and improved drought tolerance (Resmi et al., 2015). In the present study, the growth of OE lines and WT tobacco seedlings under nature drought stress was evaluated (Figure 4A). The results showed that seedlings of the OE lines showed increased plant fresh weight and root elongation under drought stress (Figures 4B,C). Exposure to drought decreased the biomass of transgenic and wild-type tobacco, but the biomass of the OE lines was higher than that of wild-type under drought stress, suggesting that *CpCHS1* increased the post-germination drought tolerance. Under drought treatment, the fresh weight of transgenic plants was significantly higher than that of WT, especially OE5 with the highest expression level. The root length of the OE line was also significantly higher than that of WT under drought treatment. These results suggest that *CpCHS1* can resist drought-induced growth inhibition by increasing root length and fresh weight of tobacco. Secondary metabolites are among the main participants in the response to abiotic stress, and mutations in some genes in the flavonoid biosynthesis pathway weaken the drought tolerance of plants and lead to a reduction in biomass accumulation (Baozhu et al., 2022). These findings suggest that flavonoid biosynthesis-related genes have crucial regulatory roles in abiotic stress.

### *CpCHS1* enhances drought tolerance in tobacco

**Figure 5:**
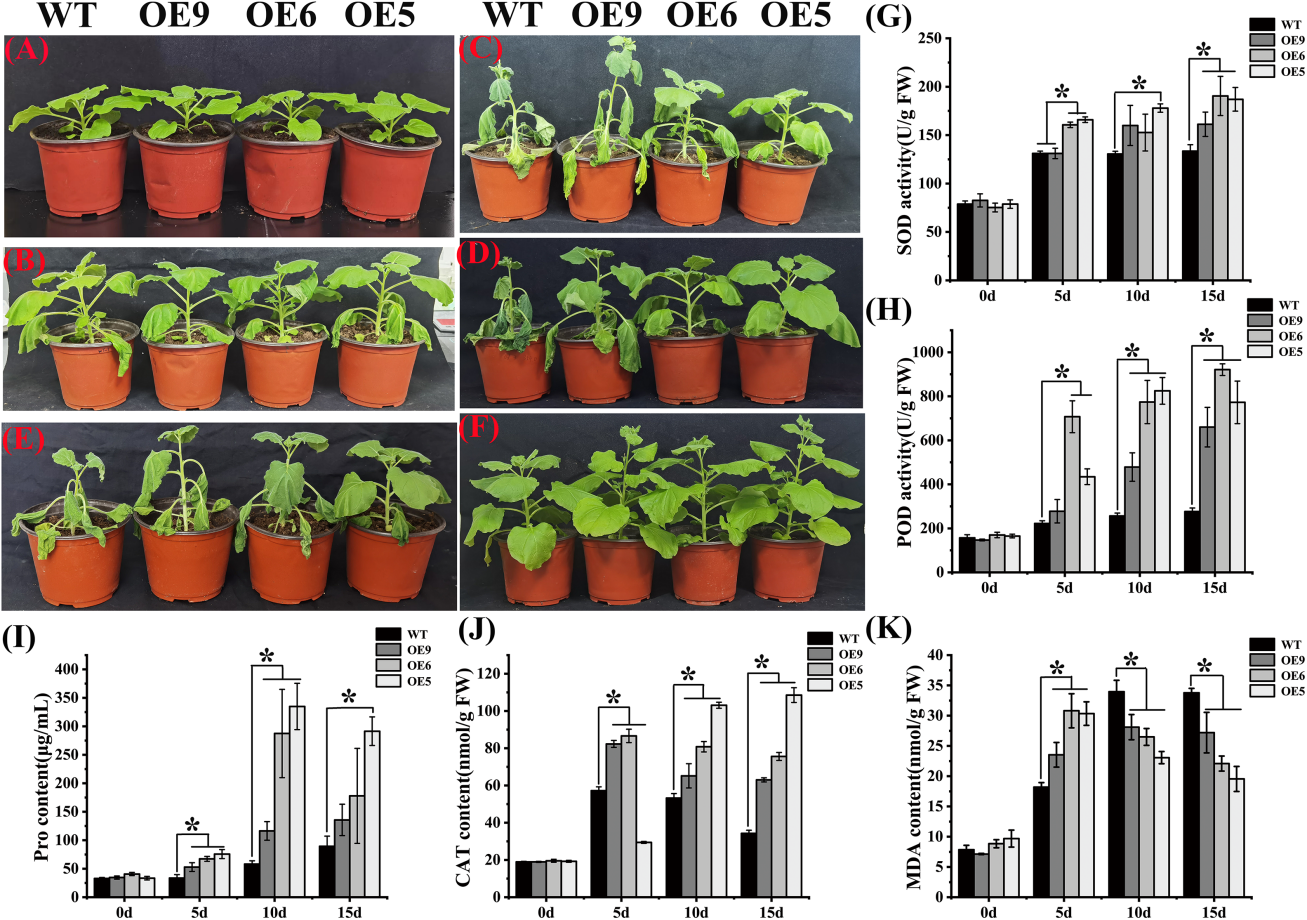
WT and OE tobacco lines are under drought treatment. **(A–D)** Phenotypes of tobacco drought treatments at 0, 5, 10, and 15 d. **(E)** Tobacco state after rehydration for 7 days after drought treatment. **(F)** Tobacco is grown normally for 15 days. **(G–K)** Determination of SOD, POD, Pro, CAT, and MDA contents in tobacco under drought treatment. * represent the significant differences of three replicates as determined by SPSS software (*p* < 0.05).

Abiotic stress stimulates plants to produce protective flavonoids, including a significant accumulation of anthocyanins, which are stress tolerance mechanisms developed by plants (Petrussa et al., 2013). Flavonoids can promote antioxidant substances such as SOD and CAT activities, to scavenge ROS produced by plants in adverse environments, thereby enhancing plant tolerance (Wang et al., 2021). In the current study, the OE lines of tobacco exhibited strong drought tolerance (Figure 5). With the increase in the duration of drought treatment, the OE line showed stronger tolerance (Figures 5A–D). At 10 days of drought treatment, the leaves of the WT had wilted, whereas those of the OE line tobacco were turgid, especially in the high expression line OE5. At 15 days of drought stress, the lines exhibiting slightly lower expression of OE9 and OE6 showed wilting of mature leaves, but the situation was slightly better for OE6. The line OE5 still behaved normally at 15 days. After rehydration for 7 days following drought treatment, the growth condition of OE lines was better than that of the WT (Figure 5E). In tobacco grown for 15 days under the non-stress condition, the plant height of the OE line was higher than that of the WT (Figure 5F). The SOD, POD, and CAT activities, and MDA and Pro contents were determined after drought treatment (Figures 5G–K). At day 0, no difference between the WT and OE lines was observed, but the SOD, POD, and CAT activities and Pro contents of the OE lines were significantly higher than those of the wild type under drought stress. The content of MDA in OE was higher than that of wild type on the 5th day of drought treatment, but with the increase in the duration of drought exposure, the content of MDA gradually decreased and was significantly lower than that of the WT after 10 days. These results indicated that overexpression of *CpCHS1* increased SOD, POD, Pro, and CAT activities and Pro content, and decreased the MDA contents during drought treatment, thereby enhancing the drought tolerance of the seedling.

CHS and related genes have been studied to varying extents in other plant species. In *Coelogyne ovalis*, the *CoCHS* gene was expressed in all tissues analyzed, up-regulated under different abiotic stresses, and positively correlated with anthocyanin accumulation (Singh and Kumaria, 2020). The expression of *SoCHS1* in *Syringa oblata* was the highest before flowering, and the transcription level was highest in the corolla lobes and calyx; overexpression of *SoCHS1* in tobacco led to a darker corolla color of flowers, suggesting that this gene may be involved in anthocyanin synthesis (Wang et al., 2017b). In poplar, *PtrCHS4* systemically responds to traumatic stress, with a 2.4-fold increase in transcript levels observed after 24 h of trauma treatment (Sun et al., 2011). Heterologous overexpression of *EaCHS1* in *Eupatorium adenophorum* in tobacco increases flavonoid accumulation downstream of CHS and promotes the up-regulated expression of related genes; maintaining ROS homeostasis during seed germination and root development regulates tobacco tolerance of salt stress (Lijuan et al., 2015). These results are consistent with those of the present study, the overexpression of *CpCHS1* overexpression increased the activities of antioxidant enzymes such as SOD and CAT, and eliminated the negative effects of drought stress (Figure 5).

**Figure 6:**
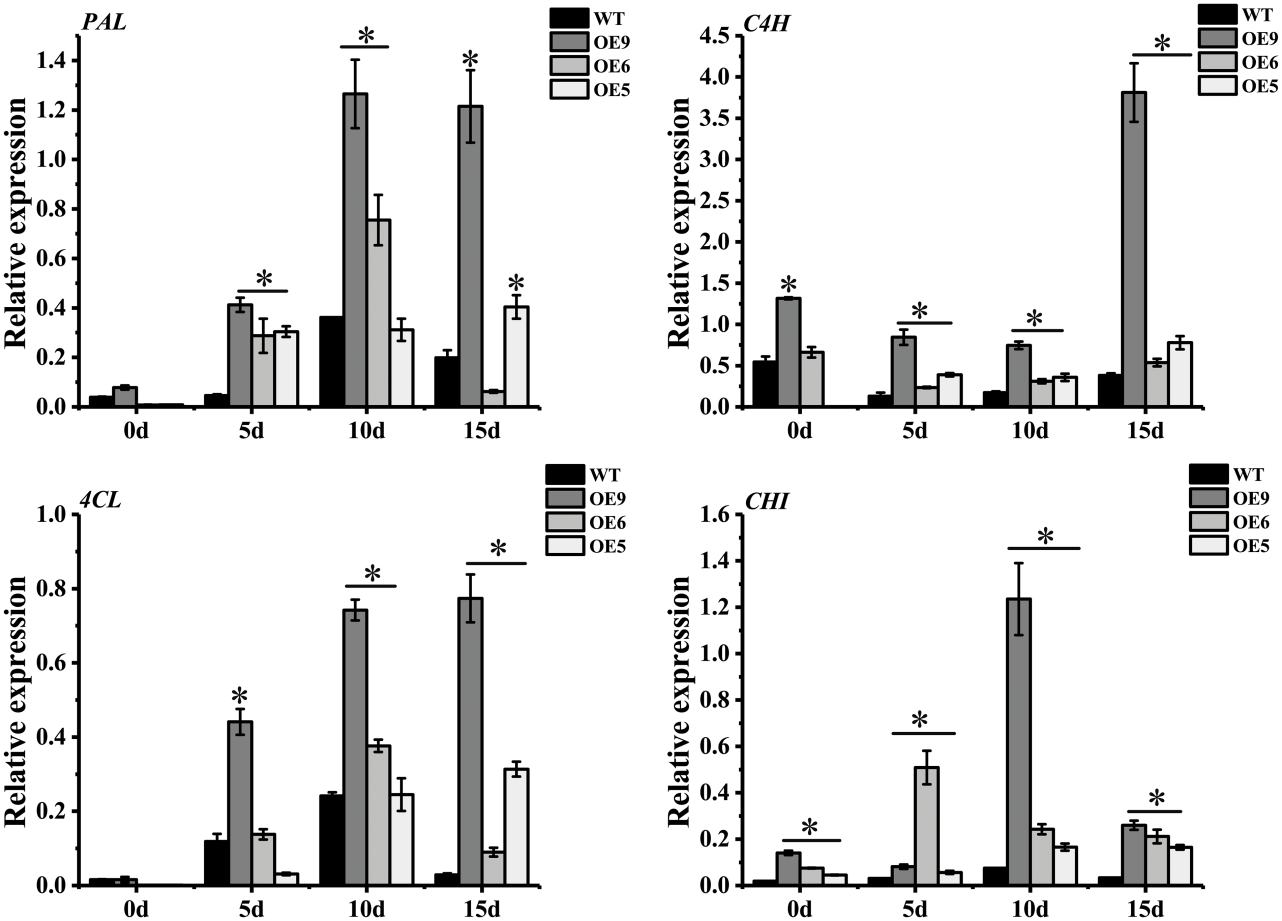
Quantitative qRT-PCR analysis of different gene expressions in WT and OE lines. *PAL*, *C4H*, *4CL* and *CHI* represent genes of phenylalanine ammonia-lyase, cinnamate-4-hydroxylase, 4-coumarate coenzyme A ligase and chalcone isomerase, respectively. Actin was used as an internal standard for the analysis. * represent the significant differences of three replicates as determined by SPSS software (*p* < 0.05).

**Figure 7:**
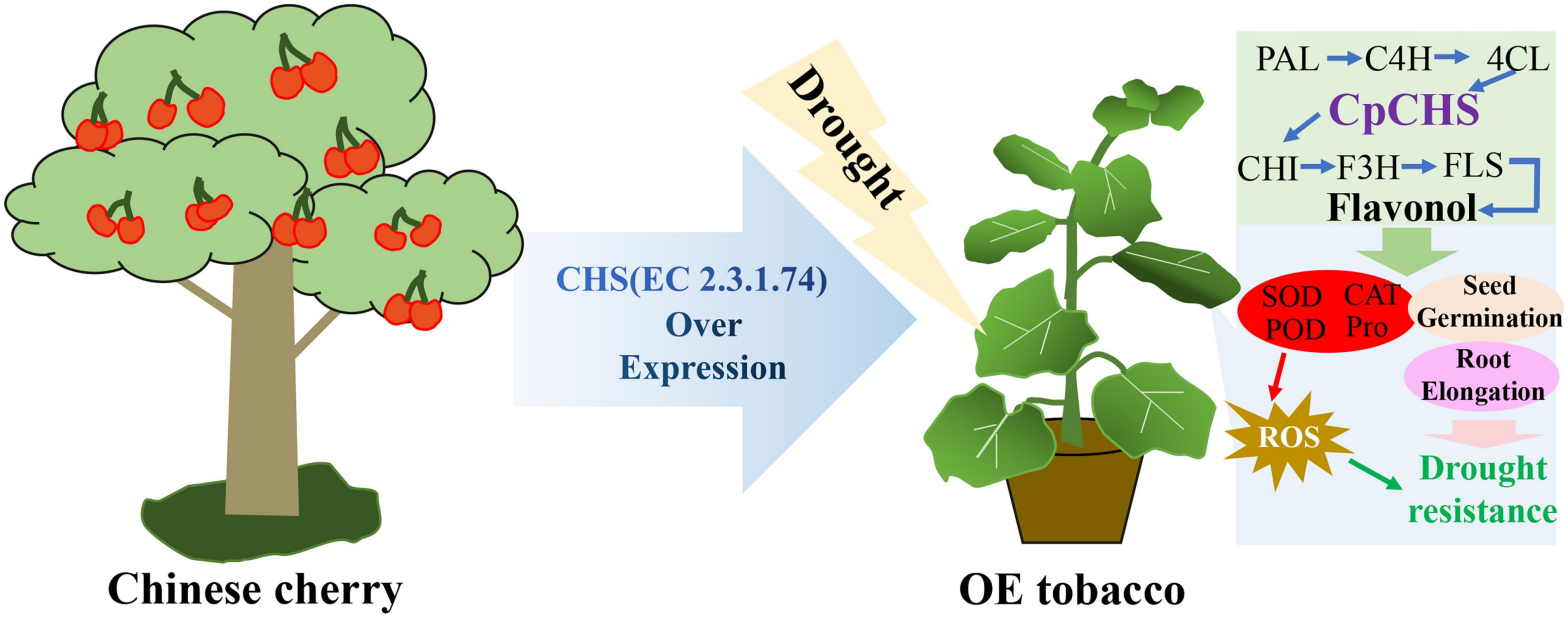
A possible model of *CpCHS1* responding to drought stress in the transgenic of tobacco. *CpCHS1* induced changes in related genes in the flavonol biosynthesis pathway under drought stress, increased SOD, POD, CAT, and Pro contents, and scavenged ROS accumulated in drought stress. In addition, the germination rate and root length of tobacco in a dehydrated environment are increased, and finally, the purpose of drought resistance is achieved.

Tomato *SlF3HL* overexpression in tobacco confers stronger tolerance of low temperature, high temperature, salt and oxidative stress, higher flavonoid content and lower MDA content than those of the wild type (Meng et al., 2015). In the current study, genes associated with the flavonol synthesis pathway were differentially expressed under drought stress. Among these genes, *PAL*, *C4H*, *4CL*, and *CHI* in the OE lines were up-regulated under drought treatment, and the expression levels were higher than those of the wild type, especially in line OE9 (Figure 6). Genes such as *PAL*, *C4H*, and *C3H* of *Asarum sieboldii* Miq are up-regulated under drought treatment, indicating the involvement of these genes in the response to drought (Liu et al., 2021a). Heterologous overexpression of *Dendrobium officinale DoFLS1* in *Arabidopsis* increases the flavonol content, and the gene was up-regulated under drought and cold stress (Yu et al., 2021). The expression of *CsF3H* in saffron is significantly enhanced under UV-B radiation, dehydration, and salt stress; overexpression of *CsF3H* in tobacco results in massive accumulation of dihydroquercetin, which confers tolerance to dehydration stress by increasing chlorophyll contents and reducing the MDA content (Baba and Ashraf, 2019). These results suggest that flavonoid synthesis-related genes play an important role in resistance to abiotic stress. In the present study, *CpCHS1*is to a crucial enzyme in the flavonoid biosynthesis pathway, and heterologous overexpression enhanced the drought resistance of tobacco. The changes in expression of flavonoid-related genes under drought stress led to an increase in the total flavonoid content, and increased activities of antioxidant enzymes, such as SOD and POD, that scavenge ROS generated under abiotic stress, thereby improving plant tolerance (Figure 7). Plants produce excess reactive oxygen species under abiotic stress and cause oxidative damage to the body. Studies have shown that plants can remove excess ROS through the transcription of related genes in the anthocyanin synthesis pathway (Wang et al., 2020a). As a key rate-limiting enzyme in flavonoid biosynthesis, CHS gene plays a critical role in plant stress resistance (Figure 7). It is worth noting that the excessive accumulation of flavonoids under experimental conditions does not bring about plant growth inhibition (Nakabayashi et al., 2014), which will also be a new direction for future breeding. This study lays a foundation for the study of flavonoid pathway-related genes in cherries and provides a reference for the selection of superior germplasm in cherries for a drought environment.

## Conclusion

In this study, three genes encoding chalcone synthase were identified from the sweet cherry genome. The three genes contained fewer introns and were evolutionarily similar to CHS genes of other members of the Rosaceae family. The tertiary structure of *PavCHS1* protein is a homodimer composed of two subunits, and the conserved catalytic sites may be located in Cys163, Phe214, His302, and Asn335. The three genes were differentially expressed in different tissues. In addition, the mature leaves of Chinese cherries contain high levels of flavonoids. Overexpression of the Chinese cherry *CpCHS1* gene in tobacco improved the seed germination frequency and the drought tolerance of seedlings exposed to drought stress (Figure 7). In addition, *CpCHS1* enhanced the drought resistance of tobacco, at least partly, by increasing the activities of SOD, POD, and CAT, increasing the Pro content, and decreasing the MDA content.

## Data availability statement

The datasets presented in this study can be found in online repositories. The names of the repository/repositories and accession number(s) can be found in the article/Supplementary material.

## Author contributions

QH, GQ, and SL conceived and designed the study. SL, CS, and YH performed most experiments. QH and ZW analyzed the data. QH and SL wrote the first draft of the manuscript. XC and GQ revised the manuscript. GQ supervised the project and reviewed the manuscript. All authors contributed to the article and approved the submitted version.

## Funding

This research was supported by National Natural Science Foundation of China (Grant nos. 32160700 and 32160701) and the Science and Technology Foundation of Guizhou Province, China (Grant no. [2020] 1Y114 and [2021] Yiban231).

## Conflict of interest

The authors declare that the research was conducted in the absence of any commercial or financial relationships that could be construed as a potential conflict of interest.

## Publisher’s note

All claims expressed in this article are solely those of the authors and do not necessarily represent those of their affiliated organizations, or those of the publisher, the editors and the reviewers. Any product that may be evaluated in this article, or claim that may be made by its manufacturer, is not guaranteed or endorsed by the publisher.

## Supplementary material

The Supplementary material for this article can be found online at: https://www.frontiersin.org/articles/10.3389/fpls.2022.989959/full#supplementary-material

## Footnotes

^1^ https://www.rosaceae.org/

^2^ https://www.expasy.org/

^3^ https://meme-suite.org/meme/

^4^ http://pfam.xfam.org/

^5^ https://www.uniprot.org/

^6^ https://swissmodel.expasy.org/

^7^ http://www.sbg.bio.ic.ac.uk/phyre2/html/page.cgi?id=index

^8^ https://www.chiplot.online/
