# Supplementary material for: Genome-wide characterization of chalcone synthase genes in sweet cherry and functional characterization of CpCHS1 under drought stress
Source: Front Plant Sci. 2022 Aug 19;13:989959. doi: 10.3389/fpls.2022.989959 (PMC9437463; doi:10.3389/fpls.2022.989959)
Supplement: Supplementary file 1 [file Data_Sheet_1.docx]

Table S1. Gene cloning, expression analysis and gene detection primers

| Primer name | Primer sequences 5’-3’ | Description |
| --- | --- | --- |
| *CpCHS1-*F | 5'-ATGGTGACCGTGGAGGAAGTTC-3’ | CDS amplification |
| *CpCHS1-*R | 5'-TCAAGCGTTCAAGCCCACG-3' |  |
| *CpCHS1-q*RT-F | 5'-GGGTGCTCGTGTTCTCGTTGTG-3' | qRT-PCR |
| *CpCHS1-q*RT-R | 5'-CGATCTCGGGAATTGGGTCTGC-3' |  |
| 35S-*CpCHS1-*F | 5'-AAGATGGACCCCCACCCACGAGGA-3' | Detection of transgenic |
| 35S-*CpCHS1-*R | 5'-GACGGATGAGCGGAGGCCCAAGAG-3' |  |
| *EF1-α-F* | 5'- AGCTTCACCTCCCAGGTCATC-3' | Tobacco reference gene |
| *EF1-α-R* | 5'-AGAACGCCTGTCAA TCTTGG-3' |  |

Table S2. CDS and protein sequences of PavCHSs and CpCHS1.

PavCHS1 (CDS)

ATGGTGACCGTGGAGGAAGTTCGCAAGGCTCAAAGGGCTGAGGGTACTGCCACTGTGTTGGCCATTGGGACAGCAACTCCTCCCAATTGTGTTGATCAGGCCACCTACCCTGACTACTACTTTCGTATCACCAACAGTGAGCACAAAGCTGAGCTTAAAAAAAAATTCCAGCGCATGTGTGACAGATCTATGATCAAGAAGCGTTATATGTACCTGACTGAAGAAATTCTAAAAGAGAACCCAAGTATGTGCGAGTACATGGCACCTTCACTTGATGCCAGGCAGGACATGGTGGTTGTTGAAATTCCAAAACTCGGCAAAGAAGCTGCCACCAAGGCCATTAAGGAATGGGGCCAGCCCAAGTCCAAAATTACGCATTTGGTCTTTTGCACCACCAGTGGTGTGGACATGCCTGGGGCCGACTATCAGCTCACTAAGCTCTTGGGCCTCCGCCCGTCCGTCAAGCGCCTCATGATGTACCAACAAGGGTGTTTCGCAGGCGGCACGGTGCTCAGGTTGGCCAAGGACTTGGCTGAGAACAACAAGGGTGCTCGTGTTCTTGTTGTGTGCTCGGAGATCACCGCGGTCACCTTCCGTGGGCCTAGTGACACCCACCTTGACAGTTTAGTGGGCCAGGCCTTATTCGGTGATGGTGCAGCAGCTATTATTGTGGGTGCGGAACCAATTCCCGAGATCGAGAAGCCGCTATTTGAAGTGGTCTCAGCAGCCCAAACCATCCTTCCCGACAGTGATGGAGCCATCGACGGGCATCTCCGTGAAGTTGGGCTTACATTTCATCTACTCAAGGATCTTCCCGGGCTTATTTCGAAGAACATTGAGAAGAGTCTGAACGAGGCCTTCCAACCTTTGGGCATCTCGGATTGGAACTCACTTTTCTGGATTGCACACCCTGGTGGCCCAGCAATTCTAGACCAAGTAGAGTCCAAATTGGCCCTGAAGCCTGAAAAATTAGAGCCCACAAGACACATACTATCTGAGTACGGAAACATGTCCAGTGCCTGTGTGTTATTTATTTTGGATGAGGTTAGGAAGAGGGCTACTAAGAAAGGACTAAAAACCACAGGAGATGGACTGGATTGGGGTGTGCTGTTTGGGTTTGGGCCTGGGCTCACTGTTGAGACCGTGGTGCTTCACAGCGTGGGCTTGAACGCTTGA

PavCHS1 (Protein)

MVTVEEVRKAQRAEGTATVLAIGTATPPNCVDQATYPDYYFRITNSEHKAELKKKFQRMCDRSMIKKRYMYLTEEILKENPSMCEYMAPSLDARQDMVVVEIPKLGKEAATKAIKEWGQPKSKITHLVFCTTSGVDMPGADYQLTKLLGLRPSVKRLMMYQQGCFAGGTVLRLAKDLAENNKGARVLVVCSEITAVTFRGPSDTHLDSLVGQALFGDGAAAIIVGAEPIPEIEKPLFEVVSAAQTILPDSDGAIDGHLREVGLTFHLLKDLPGLISKNIEKSLNEAFQPLGISDWNSLFWIAHPGGPAILDQVESKLALKPEKLEPTRHILSEYGNMSSACVLFILDEVRKRATKKGLKTTGDGLDWGVLFGFGPGLTVETVVLHSVGLNA

PavCHS1-like (CDS)

ATGGTGACCGTCGAGGAAGTTCGCAAGGCTCAACGGGCAGAGGGTCCAGCCACGGTCTTGGCCATTGGGACTTCAAATCCTCCCAATTGTGTTGATCAAGCCACATACCCTGACTACTACTTCCGTATCACCAACAGTGAGCACAAAACTGAGCTCAAAGAAAAATTCCAGCGCATGTGTGACAAATCTATGATCAAGAAGCGTTACATGTACTTGACTGAAGAAATTCTTAAAGAGAACCCAACTATGTGTGAGTACATGGCACCTTCCCTTGATGCCAGACAGGATATGGTGGTGGTTGAAATTCCAAAACTGGGAAAAGAAGCTGCTACCAAGGCCATTAAGGAATGGGGCCAACCCAAGTCCAAAATTACCCATTTGGTCTTTTGCACCACTAGTGGTGTGGACATGCCCGGGGCCGATTACCAGCTCACCAAGCTCTTGGGCCTCCGCTCATCCGTCAAGCGCCTCATGATGTACCAACAAGGCTGTTTTGCCGGCGGCACAGTGCTCCGGTTGGCCAAGGACTTGGCCGAGAACAACAGGGGTGCTCGTGTTCTCGTTGTGTGCTCGGAGATCACCGCCGTCACCTTCCGTGGGCCCAGTGATACCCACCTTGACAGTTTAGTGGGCCAGGCTTTATTTGGGGATGGTGCAGCAGCTATTATTGTGGGTGCGGACCCAATTCCCGAGATCGAGAAGCCCCTATTTGAAGTGGTCTCAGCAGCCCAAACCATCCTTCCCGACAGTGATGGAGCCATCGACGGACATCTCCGTGAAGTTGGGCTTACATTTCATCTGCTCAAGGATGTTCCCGGACTTATTTCGAAGAACATCAAGAAGAGTCTGAACGAGGCCTTCCAACCTTTGGGCATCTCGGATTGGAACTCACTATTCTGGATTGCACACCCAGGTGGCCCAGCAATTCTAGACCAAGTAGAGTCCAAATTGGCCCTGAAGCCTGAGAAATTAGAGGCTACAAGACACATACTATCTGAGTACGGAAACATGTCGAGTGCCTGTGTGCTATTTATTTTGGATGAGGTAAGGAAGAGGGCTACCAAGAAAGGACTAAAAACTACAGGAGATGGACTGGATTGGGGTGTGCTGTTTGGGTTTGGACCAGGACTCACTGTTGAGACCGTGGTGCTTCACAGCGTGGGCTTGAATGCTTGA

PavCHS1-like (Protein)

MVTVEEVRKAQRAEGPATVLAIGTSNPPNCVDQATYPDYYFRITNSEHKTELKEKFQRMCDKSMIKKRYMYLTEEILKENPTMCEYMAPSLDARQDMVVVEIPKLGKEAATKAIKEWGQPKSKITHLVFCTTSGVDMPGADYQLTKLLGLRSSVKRLMMYQQGCFAGGTVLRLAKDLAENNRGARVLVVCSEITAVTFRGPSDTHLDSLVGQALFGDGAAAIIVGADPIPEIEKPLFEVVSAAQTILPDSDGAIDGHLREVGLTFHLLKDVPGLISKNIKKSLNEAFQPLGISDWNSLFWIAHPGGPAILDQVESKLALKPEKLEATRHILSEYGNMSSACVLFILDEVRKRATKKGLKTTGDGLDWGVLFGFGPGLTVETVVLHSVGLNA

PavCHS2 (CDS)

ATGGTGACCGTGGAGGAAGTTCGCAAGGCTCAAAGGGCTGAGGGTACTGCCACTGTGTTGGCCATTGGGACAGCAACTCCTCCCAATTGTGTTGATCAGGCCACCTACCCTGACTACTACTTTCGTATCACCAACAGTGAGCACAAAGCTGAGCTTAAAAAAAAATTCCAGCGCATGTGTGACAGATCTATGATCAAGAAGCGTTACATGTACCTGACTGAAGAAATTCTAAAAGAGAACCCAAGTATGTGCGAGTACATGGCACCTTCACTTGATGCCAGGCAGGACATGGTGGTTGTTGAAATTCCAAAACTCGGCAAAGAAGCTGCCACCAAGGCCATTAAGGAATGGGCCAGCCCAAGCGGCACGGTGCTCAGGTTGGCCAAGGACTTGGCTGAGAACAACAAGGGTGCTCGTGTTCTTGTTGTGTGCTCGGAGATCACCGCGGTCACCTTCCGTGGGCCTAGTGACACCCACCTTGACAGTTTAGTGGGCCAGGCCTTATTCGGTGATGGTGCAGCAGCTATTATTGTGGGTGCAGAACCAATTCCCGAGATCGAGAAGCCGCTATTTGAAGTGGTCTCAGCAGCCCAAACCATCCTTCCCGACAGTGATGGAGCCATCGACGGGCATCTCCGTGAAGTTGGGCTTACATTTCATCTACTCAAGGATCTTCCCGGGCTTATTTCGAAGAACATTGAGAAGAGTCTGAACGAGGCCTTCCAACCTTTGGGCATCTCGGATTGGAACTCACTTTTCTGGACCCTGGTGGCCCAGCAATTCTAG

PavCHS2 (Protein)

MVTVEEVRKAQRAEGTATVLAIGTATPPNCVDQATYPDYYFRITNSEHKAELKKKFQRMCDRSMIKKRYMYLTEEILKENPSMCEYMAPSLDARQDMVVVEIPKLGKEAATKAIKEWASPSGTVLRLAKDLAENNKGARVLVVCSEITAVTFRGPSDTHLDSLVGQALFGDGAAAIIVGAEPIPEIEKPLFEVVSAAQTILPDSDGAIDGHLREVGLTFHLLKDLPGLISKNIEKSLNEAFQPLGISDWNSLFWTLVAQQF

CpCHS1 (CDS)

ATGCATGCTCGAGCGGCCGCCAGTGTGATGGATATCTGCAGAATTGCCCTTATGGTGACCGTGGAGGAAGTTCGCAAGGCTCAGCGGGCAGAGGGTCCGGCCACCGTCTTGGCCATTGGGACTGCAACTCCTCCCAATTGTGTTGATCAAGCCACCTACCCTGACTACTACTTCCGCATCACCAACAGTGAGCACAAAACTGACCTCAAAGAAAAATTCCAGCGCATGTGTGACAAATCTATGATCAAGAAGCGTTACATGTACTTGACTGAAGAAATTCTTAAAGAAAACCCAAGTATGTGTGAGTACATGGCACCTTCACTTGATGCCAGGCAGGATATGGTGGTGGTTGAAATTCCAAAACTGGGAAAAGAAGCTGCTACCAAGGCCATTAAGGAATGGGGCCAGCCTAAGTCCAAAATTACCCATTTGGTCTTTTGCACCACCAGTGGTGTGGACATGCCCGGGGCTGACTACCAACTCACCAAGCTCTTGGGCCTCCGCTCATCCGTCAAGCGGCTCATGATGTACCAACAAGGGTGTTTTGCAGGTGGCACGGTGCTCCGATTGGCCAAGGACTTGGCTGAGAACAACAGGGGTGCTCGTGTTCTCGTTGTGTGCTCGGAGATCACTGCCGTCACCTTCCGGGGTCCCAATGATACCCACCTTGACAGTTTAGTGGGCCAGGCCTTATTTGGTGATGGTGCAGCAGCTATTATTGTGGGTGCAGACCCAATTCCCGAGATCGAGAAGCCCCTATTTGAAGTGGTGTCAGCAGCCCAAACCATCCTTCCCGACAGTGATGGAGCCATCGACGGACATCTCCGTGAAGTTGGGCTTACATTTCATCTGCTCAAGGATGTTCCCGGGCTTATTTCAAAGAACATCGAGAAGAGTCTGAAGGAGGCCTTCCAACCTTTGGGCATCTCGGATTGGAACTCACTATTCTGGATTGCACACCCAGGTGGCCCAGCAATTCTAGACCAAGTAGAGTCCAAATTGGCCCTGAAGCCTGAGAAATTAGAGGCTACAAGACACATACTATCTGAGTACGGAAACATGTCGAGTGCCTGTGTGCTATTTATTTTGGATGAGGTAAGGAAGAGGGCTACCAAGAAAGGACTAAAAACCACAGGAGATGGACTGGATTGGGGTGTGCTGTTTGGGTTTGGACCAGGACTCACTGTTGAGACCGTGGTGCTTCACAGCGTGGGCTTGAACGCTTGA

CpCHS1 (Protein)

MHARAAASVMDICRIALMVTVEEVRKAQRAEGPATVLAIGTATPPNCVDQATYPDYYFRITNSEHKTDLKEKFQRMCDKSMIKKRYMYLTEEILKENPSMCEYMAPSLDARQDMVVVEIPKLGKEAATKAIKEWGQPKSKITHLVFCTTSGVDMPGADYQLTKLLGLRSSVKRLMMYQQGCFAGGTVLRLAKDLAENNRGARVLVVCSEITAVTFRGPNDTHLDSLVGQALFGDGAAAIIVGADPIPEIEKPLFEVVSAAQTILPDSDGAIDGHLREVGLTFHLLKDVPGLISKNIEKSLKEAFQPLGISDWNSLFWIAHPGGPAILDQVESKLALKPEKLEATRHILSEYGNMSSACVLFILDEVRKRATKKGLKTTGDGLDWGVLFGFGPGLTVETVVLHSVGLNA

Table S3. Sweet cherry CHS gene members information

| Gene ID in GDR | Gene Name | CDS (bp) | Protein (aa) | Chromosome Location | Molecular weight | Theoretical pI | Subcellular localization prediction | Grand average of hydropathicity (GRAVY) |
| --- | --- | --- | --- | --- | --- | --- | --- | --- |
| FUN_000072-T1 | *PavCHS1* | 1173 | 391 | chr_1: 4604449-4606446 | 42773.54 | 6.48 | Cytoplasm | -0.088 |
| FUN_000076-T1 | *PavCHS1-like* | 1173 | 391 | chr_1: 4623023-4661560 | 42778.47 | 6.47 | Cytoplasm | -0.098 |
| FUN_000075-T1 | *PavCHS2* | 783 | 261 | chr_1: 4636423-4638170 | 28755.09 | 5.38 | Cytoplasm | -0.125 |

**All sequences used in phylogeny and multiple sequence alignments in this study.**

>PavCHS2

MVTVEEVRKAQRAEGTATVLAIGTATPPNCVDQATYPDYYFRITNSEHKAELKKKFQRMCDRSMIKKRYMYLTEEILKENPSMCEYMAPSLDARQDMVVVEIPKLGKEAATKAIKEWASPSGTVLRLAKDLAENNKGARVLVVCSEITAVTFRGPSDTHLDSLVGQALFGDGAAAIIVGAEPIPEIEKPLFEVVSAAQTILPDSDGAIDGHLREVGLTFHLLKDLPGLISKNIEKSLNEAFQPLGISDWNSLFWTLVAQQF

>PavCHS1

MVTVEEVRKAQRAEGTATVLAIGTATPPNCVDQATYPDYYFRITNSEHKAELKKKFQRMCDRSMIKKRYMYLTEEILKENPSMCEYMAPSLDARQDMVVVEIPKLGKEAATKAIKEWGQPKSKITHLVFCTTSGVDMPGADYQLTKLLGLRPSVKRLMMYQQGCFAGGTVLRLAKDLAENNKGARVLVVCSEITAVTFRGPSDTHLDSLVGQALFGDGAAAIIVGAEPIPEIEKPLFEVVSAAQTILPDSDGAIDGHLREVGLTFHLLKDLPGLISKNIEKSLNEAFQPLGISDWNSLFWIAHPGGPAILDQVESKLALKPEKLEPTRHILSEYGNMSSACVLFILDEVRKRATKKGLKTTGDGLDWGVLFGFGPGLTVETVVLHSVGLNA

>PavCHS1-like

MVTVEEVRKAQRAEGPATVLAIGTSNPPNCVDQATYPDYYFRITNSEHKTELKEKFQRMCDKSMIKKRYMYLTEEILKENPTMCEYMAPSLDARQDMVVVEIPKLGKEAATKAIKEWGQPKSKITHLVFCTTSGVDMPGADYQLTKLLGLRSSVKRLMMYQQGCFAGGTVLRLAKDLAENNRGARVLVVCSEITAVTFRGPSDTHLDSLVGQALFGDGAAAIIVGADPIPEIEKPLFEVVSAAQTILPDSDGAIDGHLREVGLTFHLLKDVPGLISKNIKKSLNEAFQPLGISDWNSLFWIAHPGGPAILDQVESKLALKPEKLEATRHILSEYGNMSSACVLFILDEVRKRATKKGLKTTGDGLDWGVLFGFGPGLTVETVVLHSVGLNA

>CpCHS1

MHARAAASVMDICRIALMVTVEEVRKAQRAEGPATVLAIGTATPPNCVDQATYPDYYFRITNSEHKTDLKEKFQRMCDKSMIKKRYMYLTEEILKENPSMCEYMAPSLDARQDMVVVEIPKLGKEAATKAIKEWGQPKSKITHLVFCTTSGVDMPGADYQLTKLLGLRSSVKRLMMYQQGCFAGGTVLRLAKDLAENNRGARVLVVCSEITAVTFRGPNDTHLDSLVGQALFGDGAAAIIVGADPIPEIEKPLFEVVSAAQTILPDSDGAIDGHLREVGLTFHLLKDVPGLISKNIEKSLKEAFQPLGISDWNSLFWIAHPGGPAILDQVESKLALKPEKLEATRHILSEYGNMSSACVLFILDEVRKRATKKGLKTTGDGLDWGVLFGFGPGLTVETVVLHSVGLNA

>PyCHS

MVTVEEVRKAQRAEGPATILAIGTATPPNCVDQATYPDYYFRITNSEHKTDLKEKFQRMCDKSMIKKRYMYLTEEILKENPSMCEYMAPSLDARQDMVVVEIPKLGKEAATKAIKEWGQPKSKITHLVFCTTSGVDMPGADYQLTKLLGLRSSVKRLMMYQQGCFAGGTVLRLAKDLAENNRGARVLVVCSEITAVTFRGPNDTHLDSLVGQALFGDGAAAIIVGADPIPEIEKPLFEVVSAAQTILPDSDGAIDGHLREVGLTFHLLKDVPGLISKNIEKSLNEAFQPLGISDWNSLFWIAHPGGPAILDQVESKLALKPEKLEATRHILSEYGNMSSACVLFILDEVRKRATKKGLKTTGDGLDWGVLFGFGPGLTVETVVLHSVALNA

>PpCHS2

MVTVEEVRKAQRAEGPATVLAIGTATPPNCVDQATYPDYYFRITNSEHKTELKEKFQRMCDKSMIKKRYMYLTEEILKENPSMCEYMAPSLDARQDMVVVEIPKLGKEAATKAIKEWGQPKSKITHLVFCTTSGVDMPGADYQLTKLLGLRSSVKRLMMYQQGCFAGGTVLRLAKDLAENNRGARVLVVCSEITAVTFRGPSDTHLDSLVGQALFGDGAAAIIVGSDPIPEIEKPLFEVVSAAQTILPDSDGAIDGHLREVGLTFHLLKDVPGLISKNIEKSLNEAFQPLGISDWNSLFWIAHPGGPAILDQVESKLALKPEKLEATRHILSEYGNMSSACVLFILDEVRKRATKKGLKTTGDGLDWGVLFGFGPGLTVETVVLHSVGLNA

>PsCHS

VEEVRKAQRAEGPATVLAIGTATPPNCVDQATYPDYYFRITNSEHKAELKEKFQRMCDKSMIKKRYMYLTEEILKENPTMCEYMAPSLDARQDMVVVEIPKLGKEAATKAIKEWGQPKSKITHLVFCTTSGVDMPGADYQLTKLLGLRSSVKRLMMYQQGCFAGGTVLRLAKDLAENNRGARVLVVCSEITAVTFRGPSDTHLDSLVGQALFGDGAAAIIVGADPIPEIEKPLFEVVSAAQTILPDSDGAIDGHLREVGLTFHLLKDVPGLISKNIEKSLNEAFQPLGISDWNSLFWIAHPGGPAILDQVESKLALKPEKLEATRHILSEYGNMSSACVLFILDEVRKRATKKGLKTTGDGLDWGVLFGFGPGLTVETVVLHSVGLNA

>PdCHS

MVTVEEVRKAQRAEGPATVLAIGTATPPNCVDQATYPDYYFRITNSEHKTELKEKFQRMCDKSMIKKRYMYLTEEILKENPTMCEYMAPSLDARQDMVVVEIPKLGKEAATKAIKEWGQPKSKITHLVFCTTSGVDMPGADYQLTKLLGLRSSVKRLMMYQQGCFAGGTVLRLAKDLAENNRGARVLVVCSEITAVTFRGPNDTHLDSLVGQALFGDGAAAIIVGSDPIPEIEKPLFEVVSAAQTILPDSDGAIDGHLREVGLTFHLLKDVPGLISKNIEKSLNEAFQPLGISDWNSLFWIAHPGGPAILDQVESKLALKPEKLEATRHILSEYGNMSSACVLFILDEVRKRAAEKGLKTTGDGLDWGVLFGFGPGLTVETVVLHSVGLNA

>PaCHS

MCDKSMIKERYMYLTEEILKENPTMCEYMAPSLDARQDMVVVEIPKLGKEAATKAIKEWGQPKSKITHLVFCTTSGVDMPGADYQLTKLLGLRSSVKRLMMYQQGCFAGGTVLRLAKDLAENNRGARVLVVCSEITAVTFRGPSDTHLDSLVGQALFGDGAAAIIVGADPIPEIEKPLFEVVSAAQTILPDSDGAIDGHLREVGLTFHLLKDVPGLISKNIEKSLNEAFQPLGISDWNS

>MdCHS

MMYQQGCFAGGTVLRLAKDLAENNKGARVLVVCSEITAVTFRGPSDTHLDSLVGQALFGDGAAAVIIGADPVPEVEKPLFELVSAAQTVLPDSDGAIDGHLREVGLTFHLLKDVPGLISKNIEKSLNEALKPIGISDWNSLFWIAHPGGPAILDQVEAKLALKPEKLEATRQVLSDYGNMSSACVLFILDEVRRKSAEKGLETTGEGLEWGVLFGFGPGLTVETVVLHSVAA

>PycCHS

MVTVEEVRKAQRAEGPATVFAIGTATPPNCVDQATYPDYYFRITNSEHKTELKEKFQRMCDKSMIKTRYMYLTEEILKENPSVCEYMAPSLDARQDMVVVEVPRLGKEAATKAIKEWGQPKSKITHLVFCTTSGVDMPGADYQLTKLLGLRPSVKRLMMYQQGCFAGGTVLRLAKDLAENNKGARVLVVCSEITAVTFRGPSDTHLDSLVGQALFGDGAAAVIIGSDPVPEVEKPLFELVSAAQTILPDSDGAIDGHLREVGLTFHLLKDVPGLISKNIEKSLNEAFKPIGISDWNSLFWIAHPGGPAILDQVESKLALKPEKLEATRQVLSDYGNMSSACVLFILDEVRRKSAEKGLKTTGEGLEWGVLFGFGPGLTVETVVLHSVGA

>AtCHS

MVMAGASSLDEIRQAQRADGPAGILAIGTANPENHVLQAEYPDYYFRITNSEHMTDLKEKFKRMCDKSTIRKRHMHLTEEFLKENPHMCAYMAPSLDTRQDIVVVEVPKLGKEAAVKAIKEWGQPKSKITHVVFCTTSGVDMPGADYQLTKLLGLRPSVKRLMMYQQGCFAGGTVLRIAKDLAENNRGARVLVVCSEITAVTFRGPSDTHLDSLVGQALFSDGAAALIVGSDPDTSVGEKPIFEMVSAAQTILPDSDGAIDGHLREVGLTFHLLKDVPGLISKNIVKSLDEAFKPLGISDWNSLFWIAHPGGPAILDQVEIKLGLKEEKMRATRHVLSEYGNMSSACVLFILDEMRRKSAKDGVATTGEGLEWGVLFGFGPGLTVETVVLHSVPL

>BpCHS

SLDEIRKAQRADGPAGILAIGTANPANHVIQAEYPDYYFRITNSEHMTDLKEKFKRMCDKSTIRKRHMHLTEEFLKENPDMCAYMAPSLDARQDIVVVEVPKLGKEAAVKAIKEWGQPKSKITHVVFCTTSGVDMPGADYQLTKLLGLRPSAKRLMMYQQGCFAGGTVLRLAKDLAENNRGARVLVVCSEITAVTFRGPSDTHLDSLVGQALFSDGAAALIVGSDPDTSAGEKPIFEMVSAAQTILPDSDGAIDGHLREVGLTFHLLKDVPGLISKNIEKSLDEAFKPLGISDWNSLFWIAHPGGPAILDEVEKKLGLKAEKMRATRHVLSEYGNMSSACVLFILDEMRRKSAEDGVATTGEGLEWGVLFGFGPGL

>BoCHS

MVMCTPSSLDEIRKAQRADGPAGILAIGTANPANHVIQAEYPDYYFRITNSEHMTDLKEKFKRMCDKSTIRKRHIHLTEEFLKENPDMCAYMSPSLDARQDIVVVEVPKLGKEAAVKAIKEWGQPKSKITHVVFCTTSGVDMPGADYQLTKLLGLRPSVKRLMMYQQGCFAGGTVLRLAKDLAENNRGARVLVVCSEITAVTFRGPSDTHLDSLVGQALFSDGAAALIVGSDPDTSAGEKPIFEMVSAAQTILPDSDGAIDGHLREVGLTFHLLKDVPGLISKNIEKSLDEAFKPLGISDWNSLFWIAHPGGPAILDEVEKKLGLKAEKMRATRHVLSEYGNMSSACVLFILDEMGRKSAEDGVATTGEGLEWGVLFGFGPGLTVETVVLHSVP

>BnCHS

MVMGPSSLDEIRKAQRADGPAGILAIGTANPANHVLQAEYPDYYFRITNSEHMTDLKEKFKRMCDKSTIRKRHMHLTEEFLKENPNMCAYMAPSLDARQDLVVVEVPKLGKDAAVKAIKEWGQPKSKITHVVFCTTSGVDMPGADYQLTKLLGLRPSVKRLMMYQQGCFAGGTVLRLAKDLAENNRGARVLVVCSEITAVTFRGPSDTHLDSLVGQALFSDGAAALIVGSDPDISAGEKPIFEMVSAAQTILPDSDGAIDGHLREVGLTFHLLKDVPGLISKNIEKSLDEAFKPLGISDWNSLFWIAHPGGPAILDEVEKKLGLKAEKMRATRHVLSEYGNMSSACVLFILDEMRRKSAEDGVATTGEGLEWGVLFGFGPGLTVETVVLHSVPL

>MiCHS

MVMGATSLDEIRKAQRADGPAGILGIGTANPANHVIQAEYPDYYFRITNSEHMTDLKEKFQRMCDKSMIRKRHMHLTEDFLKENPNMCAYMAPSLDARQDIVVVEVPKLGKEAAVRAIKEWGQPKSKITHLVFCTTSGVDMPGADYQLTKLLGLRPSVKRLMMYQQGCFAGGTVLRLAKDLAENNRGARVLVVCSEITAVTFRGPSDTHLDSLVGQALFSDGAAALIVGSDPDTSVGEKPIFEMVSAAQTILPDSDGAIDGHLREVGLTFHLLKDVPGLISKNIEKSLEEAFKPLGISDWNSLFWIAHPGGPAILDQVEIKLGLKAEKMRATRHVLSEYGNMSSACVLFILDEMRKKSAQDGVATTGEGLEWGVLFGFGPGLTVETVVLRSVPL

>PcCHS

MVMGTSPPLDEIRKAQRADGPAGILSIGTANPANYVIQAEYPDYYFRITNSEHMTDLKEKFKRMCDKSMIRKRHMHLTEDFLKENPDMCAYMAPSLDARQDIVVVEVPKLGKEAAVKAIKEWGQPKSKITHVVFCTTSGVDMPGADYQLTKLLGLRPSVKRLMMYQQGCFAGGTVLRLAKDLAENNRGARVLVVCSEITAVTFRGPSDTHLDSLVGQALFSDGAAALIVGSDPDTSAGEKPIFEMV

>RsCHS

MVGTTSSLDEIRKAQRADGPAGILAIGTANPANHVLQAEYPDYYFRITNSEHMTDLKEKFKRMCDKSTIRKRHMHLTEEFLKENPNMCAYMAPSLDARQDIVVVEVPKLGKEAAVKAIKEWGQPKSKITHVVFCTTSGVDMPGADYQLTKLLGLRPSVKRLMMYQQGCFAGGTVLRLAKDLAENNRGARVLVVCSEITAVTFRGPSDTHLDSLVGQALFSDGAAALIVGSDPDVSAGEKPIFEMVSAAQTILPDSDGAIDGHLREVGITFHLLKDVPGLISKNIEKSLDEAFKPLGISDWNSLFWIAHPGGPAILDDVEKKLGLKAEKMRATRHVLSEYGNMSSACVLFILDEMRRKSLDDGVATTGEGLEWGVLFGFGPGLTVETVVLHSVPV

>OvCHS

SLDEIRKAQRADGPAGILAIGTANPANHVIQAEYPDYYFRITNSEHMTDLKEKFKRMCDKSMIRKRHMHLTEEFLKENPNMCAYMAPSLDARQDIVVVEVPKLGKEAAVKAIKEWGQPKSKITHVVFCTTSGVDMPGADYQLTKLLGLRPSVKRLMMYQQGCFAGGTVLRLAKDLAENNRGARVLVVCSEITAVTFRGPSDTHLDSLVGQALFSDGAAALIVGSDPDISAGEKPIFEMVSAAQTILPDSDGAIDGHLREVGLTFHLLKDVPGLISKNIEKSLDEAFKPLGISDWNSLFWIAHPGGPAILDQVEIKLGLKAEKMRATRHVLSEYGNMSSACVLFILDEMRRKSAEDGVATTGEGLEWGVLFGFGPGL

>OsCHS1

MAAAVTVEEVRRAQRAEGPATVLAIGTATPANCVYQADYPDYYFRITKSEHMVELKEKFKRMCDKSQIRKRYMHLTEEILQENPNMCAYMAPSLDARQDIVVVEVPKLGKAAAQKAIKEWGQPRSRITHLVFCTTSGVDMPGADYQLAKMLGLRPNVNRLMMYQQGCFAGGTVLRVAKDLAENNRGARVLAVCSEITAVTFRGPSESHLDSMVGQALFGDGAAAVIVGSDPDEAVERPLFQMVSASQTILPDSEGAIDGHLREVGLTFHLLKDVPGLISKNIERALGDAFTPLGISDWNSIFWVAHPGGPAILDQVEAKVGLDKERMRATRHVLSEYGNMSSACVLFILDEMRKRSAEDGHATTGEGMDWGVLFGFGPGLTVETVVLHSVPITAGAAA

>OsCHS2

MVTSTVKLEEVRRMQRAEGMAAVLAIGTATPANCVYQTDYPDYYFRVTNSEHLTNLKERFQRMCESSQIRKRYTHLTEEILQENPSMCVFTAPSLDARQDMVVAEVPKLGKAAAEEAIKEWGQPMSRITHLVFCTTNGVDMPGADYQVAKMLGLPTSVKRLMMYQQGCFAGGTVLRVAKDLAENNRGARVLVVCSEIMAMAFRGPSESHLDSLVGHALFGDGAAAVIVGSDPDEAADERPLFQIVSASQTILPGTEDAIVGHLREVGLTFHLPKDVPEFISDSVEGALTDAFMPLGVHDWNSIFWVVHPGGPAILDQVEEKVALHKARMRASRNVLSEYGNMASATVLFVLDEMRKLSADDGHATTGEGMDWGVLFGFGPGLTVETIVLHSVPITAAAPLIMQ

>TaCHS

MAATMTVEEVRKAQRAEGPATVLAIGTATPANCVYQADYPDYYFKITKSDHMADLKEKFKRMCDKSQIRKRYMHLTEEILQDNPNMCAYMAPSLDARQDIVVVEVPKLGKAAAQKAIKEWGQPRSKITHLVFCTTSGVDMPGADYQLTKMLGLRPSVKRLMMYQQGCFAGGTVLRLAKDLAENNRGARVLVVCSEITAVTFRGPHESHLDSLVGQALFGDGAAAVIIGADPDESIERPLFQLVSASQTILPDSEGAIDGHLREVGLTFHLLKDVPGLISKNIERALEDAFKPLGIDDWNSVFWIAHPGGPAILDMVEAKVNLNKERMRATRHVLSEYGNMSSACVLFIMDEMRKRSAEDGHSTTGEGMDWGVLFGFGPGLTVETVVLHSVPVTA

>ZmWHP1

MAGATVTVDEVRKGQRATGPATVLAIGTATPANCVYQADYPDYYFRITKSDHLTDLKEKFKRMCDKSMIRKRYMHLTEEFLSENPSMCAYMAPSLDARQDVVVTEVPKLGKAAAQEAIKEWGQPKSRITHLVFCTTSGVDMPGADYQLTKALGLRVVNRLMMYQQGCFAGGTVLRVAKDVAENNRGARVMVVCSEITAVTFRGPSESHVDSLVGQALFGDGAAAGRGGADPDGRVERPLFQLVSAAQTILPDSEGAIDGHLREVGLAFHLLKDVPGLISKNIERALEDAFEPLGISDWNSIFWVAHPGGPAILDQVEAKVGLDKARMRATRHVLSEYGNMSSACVLFILDEMRKRPAEDGQSTTGEGLDWGVLFGFGPGLTVETVVLHSVPITTGAPTAA

>ZmCHS-C2

MAGATVTVEEVRKAQRATGPATVLAIGTATPANCVYQADYPDYYFRITKSEHLTDLKEKFKRMCDKSMIRKRYMHLTEEFLAENPSMCAYMAPSLDARQDVVVVEVPKLGKAAAQKAIKEWGQPKSRITHLVFCTTSGVDMPGADYQLTKALGLRPSVNRLMMYQQGCFAGGTVLRVAKDLAENNRGARVLVVCSEITAVTFRGPSESHLDSLVGQALFGDGAAAVVVGADPDDRVERPLFQLVSAAQTILPDSEGAIDGHLREVGLTFHLLKDVPGLISKNIGRALDDAFKPLGISDWNSIFWVAHPGGPAILDQVEAKVGLDKARMRATRHVLSEYGNMSSACVLFILDEMRKRSAEDGQATTGEGLDWGVLFGFGPGLTVETVVLHSVPITTGAATA

>ZmCHS3

MWSVVDSYTVPNEAHRYGIIGHAIFGDGAGAVVVGSRPQEPVERPIFEMVSASQATVPRSERAVAAELTNCGLEYRLEFGELAAEVGDNIERCLINTMSPLGIGSVGWNNLFWVVHPGGPRIMDTFTTALRLEPEKLAASRQVFSEYGNMVGPTLIFVLDEVIRRRQQDHEEGSCEWGFMVGFGPGFTIDVMALHACTTPTPRLK

>ZmCHS8

MTVTEVPKLAARAAAKAIAEWGRPATDITHVVFSTYSGARSPSADLRLASLLGLRPSVCRTTLSLHGCSGGVRALHLAKDIAENNHGARVLVVCAEVSLISYSGPTDGCVDSVLGPALFGDGAGAVILGAGPVSGSERPLFETVCAAQTTVPMTEAAITTQFAPGGMEYRIGKQVPGIVEQTIRQCLLDVTGPLGVDVVTWNDLFWAVHCGGRAILDSVEAALGLGSQKLAASRHVLREYGNMSSAAVVFVLEEVHRRLTSKNPDGETAEWGVMVAFGPGVTVEIMVLRATTNLEEK

>HvCHS1

MAATMTVEEVRNAQRAEGPATVLAIGTATPANCVYQADYPDYYFKITKSDHMADLKEKFKRMCDKSQIRKRYMHLTEEILEENPNMCAYMAPSLDARQDIVVVEVPKLGKAAAQKAIKEWGQPRSKITHLVFCTTSGVDMPGADYQLTKMLGLRPSVKRLMMYQQGCFAGGTVLRLAKDLAENNRGARVLVVCSEITAVTFRGPHESHLDSLVGQALFGDGAAAVIIGADPDLSVERPLFQLVSASQTILPDSEGAIDGHLREVGLTFHLLKDVPGLISKNIERALEEAFKPLGIDHWNSVFWIAHQGGPAILDMVEAKVNLNKERMRATRHVLSEYGNMSSACVLFIMDEMRKRSAEDGHATTGEGMDWGVLFGFGPGLTVETVVLHSVPISAGATA

>HvCHS2

MAAVRLKEVRMAQRAEGLATVLAIGTAVPANCVYQATYPDYYFRVTKSEHLADLKEKFQRMCDKSMIRKRHMHLTEEILIKNPKICAHMETSLDARHAIALVEVPKLGQGAAEKAIKEWGQPLSKITHLVFCTTSGVDMPGADYQLTKLLGLSPTVKRLMMYQQGCFGGATVLRLAKDIAENNRGARVLVVCSEITAMAFRGPCKSHLDSLVGHALFGDGAAAAIIGADPDQLDEQPVFQLVSASQTILPESEGAIDGHLTEAGLTIHLLKDVPGLISENIEQALEDAFEPLGIHNWNSIFWIAHPGGPAILDRVEDRVGLDKKRMRASREVLSEYGNMSSASVLFVLDVMRKSSAKDGLATTGEGKDWGVLFGFGPGLTVETLVLHSVPVPVPTAASA

>SbCHS1

MAGATVTVEEVRKAQRATGPATVLAIGTATPANCVHQADYPDYYFRITKSEHMTELKEKFKRMCDKSQIRKRYMHLTEEYLAENPNMCAYMAPSLDARQDIVVVEVPKLGKAAAQKAIKEWGQPKSKITHLVFCTTSGVDMPGADYQLTKMLGLRPSVNRLMMYQQGCFAGGTVLRVAKDLAENNRGARVLVVCSEITAVTFRGPSESHLDSMVGQALFGDGAAAVIVGADPDERVERPLFQLVSASQRILPDSEGAIDGHLREVGLTFHLLKDVPGLISKNIERALEEAFKPLGITDYNSIFWVAHPGGPAILDQVEAKVGLEKERMRATRHVLSEYGNMSSACVLFILDEMRKRSAEDGQTTTGEGFDWGVLFGFGPGLTVETVVLHSVPITTGAAITA

>SbCHS2

MAGATVTVEEVRKAQRATGPATVLAIGTATPANCVHQADYPDYYFRITKSEHMTELKEKFKRMCDKSQIRKRYMHLTEEYLAENPNMCAYMAPSLDARQDIVVVEVPKLGKAAAQKAIKEWGQPKSKITHLVFCTTSGVDMPGADYQLTKMLGLRPSVNRLMMYQQGCFAGGTVLRVAKDLAENNRGARVLVVCSEITAVTFRGPSESHLDSMVGQALFGDGAAAVIVGADPDERVERPLFQLVSASQTILPDSEGAIDGHLREVGLTFHLLKDVPGLISKNIERSLEEAFKPLGITDYNSIFWVAHPGGPAILDQVEAKVGLEKERMRATRHVLSEYGNMSSACVLFILDEMRKRSAEDGRATTGEGFEWGVLFGFGPGLTVETVVLHSVPITTGAAITA

>NtCHS

MVTVEEFRRAQCAEGPATVMAIGTATPSNCVDQSTYPDYYFRITNSEHKVELKEKFKRMCEKSMIKKRYMHLTEEILKDNPNICAYMAPSLDARQDIVVVEVPKLGKEAAQKAIKEWGQPKSKITHLVFCTTSGVDMPGCDYQLTKLLGLRPSVKRFMMYQQGCFAGGTVLRMAKDLAENNKGARVLVVCSEITAVTFRGPNDTHLDSLVGQALFGDGAAAVIIGSDPIPEVERPLFELVSAAQTLLPDSEGAIDGHLREVGLTFHLLKDVPGLISKNIEKSLVEAFQPLGISDWNSLFWIAHPGGPAILDQVELKLGLKQEKLKATRKVLSNYGNMSSACVLFILDEMRKASAKEGLGTTGEGLEWGVLFGFGPGLTVETVVLHSVAT

>SmCHS

MVTVEEYRKAQRAEGPATVMAIGTSTPSNCVDQSAYPDYYFRITKSEHKTELKEKFKRMCEKSMIKKRYMYLTEEILKEKSNMCAYMAPSLDARQDIVVVEVPKLGKEAAQKAIKEWGQPKSKITHLVFCTTSGVDMPGCDYQLTKLLGLRPSVKRLMMYQQGCFAGGTVLRLAKDLAENNKGARVLVVCSEITAVTFRGPSESHLDSLVGQALFGDGAAAVIIGSDPISGVERPLFELVSAAQTLLPDSEGAIDGHLREVGLTFHLLKDVPGIISKNIDKSLVEAFQPLGISDWNSLFWIAHPGGPAILDQVELKLGLNPEKLRATREVLSNYGNMSSACVLFILDEMRKASAKQGLPTTGEGLQWGVLFGFGPGLTVETLVLHSVAA

>SlCHS1

MVTVEEYRKAQRAEGPATILAIGTSTPSNCVDQSTYPDYYFRITNSEHKTELKEKFKRMCDKSMIKKRYMHLTEEILKENPNMCAYMAPSLDARQDIVVVEVPKLGKRGTQKAIKEWGQPKSKITHLVFCTTSGVDMPACDYQLAKLLPVRPSVKRLMMYQQGCFAGGTVLRLAKDLAENNKGARVLVVCSEITAVTFRGPSESHLDSLVGQALFGDGAAAIIIGSDPIIGVERPLFELVSAAQTLVPDSEGAIDGHLREVGLTFHLLKDVPGLISKNIEKSLLEAFQPLGISDWNSLFWIAHPGGPAILDQVELKLGLKPEKLRATREVLSNYGNMSSACVLFILDEMRKASTKEGLGTTGEGLEWGVLFGFGPGLTVETVVLHSVAA

>SlCHS2

MVTVEEVRRAQRAKGPATIMAIGTATPSNCVDQSTYPDYYFRITNSEHMTELKEKFKRMCDKSMINKRYMHLTEEILKENPNICEYMAPSLDARQDIVVVEVPKLGKEAAQKAIKEWGQPKSKITHVVFCTTSGVDMPGADYQLTKLLGLRPSVKRLMMYQQGCFAGGTVIRLAKDLAENNKGARVLVVCSEITAVTFRGPSDTHLDSMVGQALFGDRAAAMIIGSDPLPEVERPLFELVSAAQTLLPDSEGAIDGHLREVGLTFHLLKDVPGLISKNIEKSLIEAFQPLGISDWNSIFWIAHPGGPAILDQVELKLSLKPEKLRATRQVLSDYGNMSSACVLFILDEMRKASSKEGLSTTGEGLDWGVLFGFGPGLTVETVVLHSVST

>CaCHS

MVTVEEVRREQRAKGPATIMAIGTATPSNCVDQATYPDYYFRITNSEHMTELKEKFQRMCDKSMIKKRYMHLTEEILKENPNICEYMAPSLDARQDIVVVEIPKLGKEAAKKAIKEWGQPKSKITHLVFCTTSGVDMPGADYQLTKLLGLRPSVKRLMMYQQGCFAGGTVIRLAKDLAENNKGARVLVVCSEITAVTFRGPSDTHLDSMVGQALFGDGAAALIVGSDPLPEVERPLFELVSAAQTLLPDSEGAIDGHLREVGLTFHLLKDVPGLISKNIEKSLIEAFQPLGNSDWNSIFWIAHPGGPTILDQVELKLGLKLEKLRATRQVLSDYGNMSRACVLFILDEMRKASAKEGLNTTGEGLDWGVLFGFGPGLTVETVVLHS

>StCHS2

MVTVEEVRKAQRAKGPATIMAIGTATPSNCVDQSTYPDYYFRITNSEHMTELKEKFKRMCDKSMINKRYMHLTEEILKENPNICEYMAPSLDARQDIVVVEVPKLGKEAAQKAIKEWGQPKSKITHVVFCTTSGVDMPGADYQLTKLLGLRPSVKRLMMYQQGCFAGGTVIRLAKDLAENNKGARVLVVCSEITAVTFSGPSDTHLDSMVGQALFGDGAAAMIIGSDPLPEVERPLFELVSAAQTLLPDSEGAIDGHLREVGLTFHLLKDVPGLISKNIEKSLIEAFQPLGISDWNSIFWIAHPGGPAILDQVELKLGLKPEKLQATRQVLSDYGNMSSACVLFILDEMRKASSKEGLSTTGEGLDWGVLFGFGPGLTVETVVLHSVST

>StCHS1B

MVTVEEYRKAQRAEGPATILAIGTSTPSNCVDQSTYPDYYFRITNSEHKTELKEKFKRMCDKSMIKKRYMHLTEEILKENPNMCAYMAPSLDARQDIVVVEVPKLGKEAAQKAIKEWGQPKSKITHLVFCTTSGVDMPGCDYQLAKLLGLRPSVKRLMMYQQGCFAGGTVLRLAKDLAENNKGARVLVVCSEITAVTFRGPSESHLDSLVGQALFGDGAAAIIMGSDPIIGVERPLFELVSAAQTLVPDSEGAIDGHLREVGLTFHLLKDVPGLISKNIEKSLLEAFQPLGISDWNSLFWIAHPGGPAILDQVELKLGLKQEKLRATREVLSNYGNMSSACVLFILDEMRKASTKEGLGTTGEGLEWGVLFGFGPGLTVETVVLHSVAT

>StCHS1A

MVTVEEYRKAQRAEGPATILAIGTSTPSNCVDQSTYPDYYFRITNSEHKTELKEKFKRMCDKSMIKKRYMHLTEEILKENPNMCAYMAPSLDARQDIVVVEVPKLGKEAAQKAIKEWGQPKSKITHLVFCTTSGVDMPGCDYQLAKLLGLRPSVKRLMMYQQGCFVGGTVLRLAKDLAENNKGARVLVVCSEITAVTFRGPSESHLDSLVGQALFGDGAAAIIMGSDPIIGVERPLFELVSAAQTLVPDSEGAIDGHLREVGLTFHLLKDVPGLISKNIEKSLLEAFQPLGISDWNSLFWIAHPGGPAILDQVELKLGLKQEKLRATREVLSNYGNMSSACVLFILDEMRKASTNEGLGTTGEGLEWGVLFGFGPGLTVETVVLHSVAT

>NaCHS

MVTVEEFRRAQRAEGPATVMAIGTATPSNCVDQSTYPDYYFRITNSEHKTELKEKFKRMCEKSMIKKRYMHLTEEILKENPNICAYMAPSLDARQDIVVVEVPKLGKEAAQKAIKEWGQPKSKITHLVFCTTSGVDMPGCDYQLTKLLGLRPSVKRFMMYQQGCFAGGTVLRMAKDLAENNKGARVLVVCSEITAVTFRGPNDTHLDSLVGQALFGDGAAAIIIGSDPIPEVERPLFELVSAAQTLLPDSEGAIDGHLREVGLTFHLLKDVPGLISKNIEKSLVEAFQPLGISDWNSLFWIAHPGGPAILDQVELKLGLKQEKLKATRNVLSNYGNMSSACVLFILDEMRKASAKEGLGTTGEGLEWGVLFGFGPGLTVETVVLHSVAT
